# Neuroimaging, Urinary, and Plasma Biomarkers of Treatment Response in Huntington’s Disease: Preclinical Evidence with the p75^NTR^ Ligand LM11A-31

**DOI:** 10.1007/s13311-021-01023-8

**Published:** 2021-03-30

**Authors:** Danielle A. Simmons, Brian D. Mills, Robert R. Butler III, Jason Kuan, Tyne L. M. McHugh, Carolyn Akers, James Zhou, Wassim Syriani, Maged Grouban, Michael Zeineh, Frank M. Longo

**Affiliations:** 1 Department of Neurology and Neurological Sciences, Stanford University School of Medicine, Stanford, CA 94305 USA; 2 Department of Radiology, Stanford University Medical Center, Stanford, CA 94305 USA

**Keywords:** Magnetic resonance imaging, Neurotrophin, p75^NTR^, Cytokine, Biomarker

## Abstract

Huntington’s disease (HD) is caused by an expansion of the CAG repeat in the huntingtin gene leading to preferential neurodegeneration of the striatum. Disease-modifying treatments are not yet available to HD patients and their development would be facilitated by translatable pharmacodynamic biomarkers. Multi-modal magnetic resonance imaging (MRI) and plasma cytokines have been suggested as disease onset/progression biomarkers, but their ability to detect treatment efficacy is understudied. This study used the R6/2 mouse model of HD to assess if structural neuroimaging and biofluid assays can detect treatment response using as a prototype the small molecule p75^NTR^ ligand LM11A-31, shown previously to reduce HD phenotypes in these mice. LM11A-31 alleviated volume reductions in multiple brain regions, including striatum, of vehicle-treated R6/2 mice relative to wild-types (WTs), as assessed with *in vivo* MRI. LM11A-31 also normalized changes in diffusion tensor imaging (DTI) metrics and diminished increases in certain plasma cytokine levels, including tumor necrosis factor-alpha and interleukin-6, in R6/2 mice. Finally, R6/2-vehicle mice had increased urinary levels of the p75^NTR^ extracellular domain (ecd), a cleavage product released with pro-apoptotic ligand binding that detects the progression of other neurodegenerative diseases; LM11A-31 reduced this increase. These results are the first to show that urinary p75^NTR^-ecd levels are elevated in an HD mouse model and can be used to detect therapeutic effects. These data also indicate that multi-modal MRI and plasma cytokine levels may be effective pharmacodynamic biomarkers and that using combinations of these markers would be a viable and powerful option for clinical trials.

**Supplementary Information:**

The online version contains supplementary material available at 10.1007/s13311-021-01023-8.

## Introduction

Huntington’s disease (HD) is a fatal neurodegenerative disorder that manifests clinically with motor impairments, cognitive deficits, and psychiatric symptoms. It is caused by a mutation in the exon 1 region of the huntingtin (HTT) gene resulting in an expansion of the polyglutamine region of the mutant huntingtin (mHtt) protein. Prominent degenerative changes in HD include early pathology in the striatum, including intranuclear mHtt aggregates, neuroinflammation, iron accumulation, and eventually neuronal loss [1–3]. With disease progression, neurodegeneration is more pervasive, extending beyond the striatum to the cortex and other subcortical regions as well as white matter [3, 4]. Therapies capable of slowing disease progression and thereby delaying or preventing symptom onset are not yet available to HD patients.

Key mechanisms underlying HD neurodegeneration involve mHtt-induced loss of neurotrophic support which can be largely attributed to brain-derived neurotrophic factor (BDNF) down-regulation and disrupted signaling via the neurotrophin receptors (NTR) tropomyosin receptor kinase B (TrkB) and p75^NTR^ [5]. Dysfunctional p75^NTR^ signaling critically contributes to dendritic spine loss as well as corticostriatal and hippocampal plasticity deficits that occur in HD mouse models [6–9]. p75^NTR^ levels are elevated in the striatum and hippocampus, but not the cortex, of HD patients and in all three brain areas of multiple HD mouse models [7, 10–13]. Targeting p75^NTR^ signaling has therefore emerged as a promising HD therapeutic strategy [5, 9]. Accordingly, our laboratory has developed an orally bioavailable small molecule p75^NTR^ ligand, LM11A-31, which selectively binds to the receptor and functions as a modulator to promote trophic signaling while abating degenerative signaling [14, 15]. In HD mouse models (R6/2 and BACHD), LM11A-31 normalized aberrant p75^NTR^ signaling. This effect was accompanied by improved motor and cognitive abilities as well as a reduction in characteristic HD pathologies, including intranuclear mHtt aggregates, dendritic spine loss, and neuroinflammation. Notably, LM11A-31 also increased the survivability of R6/2 mice [8]. These preclinical results position LM11A-31 as ripe for HD clinical testing since the compound is currently in a phase 2a clinical trial for Alzheimer’s disease (ClinicalTrials.gov: NCT03069014).

Eventual HD clinical testing of LM11A-31 and development of other HD disease-modifying therapeutics would benefit from non-invasive biomarkers that are translatable from mouse-to-human and can detect a disease-relevant biological response to putative treatments. Our previous efforts to address this need revealed that positron emission tomography (PET) imaging with [^18^F]PBR06, a radiotracer targeting translocator protein 18 kDa (TSPO) to detect microglial activation, discerned the reductive effect of LM11A-31 on neuroinflammation in the brains of multiple HD mouse models [16]. The PET imaging results are encouraging, but combining multiple biomarkers with specific and complementary advantages will further contribute to preclinical therapeutic screening and clinical trials. As HD is an inherited autosomal dominant disease, genetic testing can establish the presence of the mutation years before motor symptom onset, and many clinical, biochemical, and neuro-/molecular imaging biomarkers have been validated as potential biomarkers of disease state and/or progression (for reviews, see [17–19]). However, few, if any, of these identified biomarkers have been interrogated for their ability to discern disease-modifying treatment response in HD patients or animal models, a requisite for translational purposes. Thus, this study aimed to establish whether one or more of the biomarkers previously shown to track disease progression in HD patients and/or other neurodegenerative diseases can discern therapeutic effects in an HD mouse model using LM11A-31 as a prototype therapy.

In choosing biomarkers to investigate in this study, preference was given to those that are readily translatable from preclinical to clinical studies, applicable to a broad range of therapeutic strategies, and minimally invasive [e.g., magnetic resonance imaging (MRI), blood/urine collection]. MRI is a modality with the potential to be translatable from mouse studies to human clinical trials. Gray and white matter changes in the basal ganglia detected via structural MRI have been heralded as one of the most powerful discriminators between pre-symptomatic HD patients and healthy controls [20]. This study investigated whether LM11A-31 could prevent volume loss and connectivity changes in pre-defined brain regions of HD mice and if this effect could be detected with structural or diffusion MRI techniques. We examined other potential biomarkers for their ability to detect LM11A-31 effects, including cytokines in plasma and p75^NTR^ extracellular domain (ecd) levels in urine. Both of these fluid biomarkers have been used to evaluate disease progression in HD and/or other neurodegenerative conditions, including amyotrophic lateral sclerosis (ALS) and Alzheimer’s disease [21, 22]. To our knowledge, very few studies have used a comprehensive multi-modal *in vivo* neuroimaging approach paired with a contemporaneous examination of fluid biomarkers in an HD mouse model. Even fewer have utilized this strategy to empirically test its efficacy in elucidating the treatment response of potential HD therapeutics. Using this approach and machine learning techniques, we found that multi-modal *in vivo *MRI and levels of plasma cytokines and urinary p75^NTR^-ecd may be effective pharmacodynamic biomarkers preclinically and have a high potential for translation to HD clinical trials, with combinations of these markers as the most powerful option.

## Methods

### Study Design

**Fig. 1:**
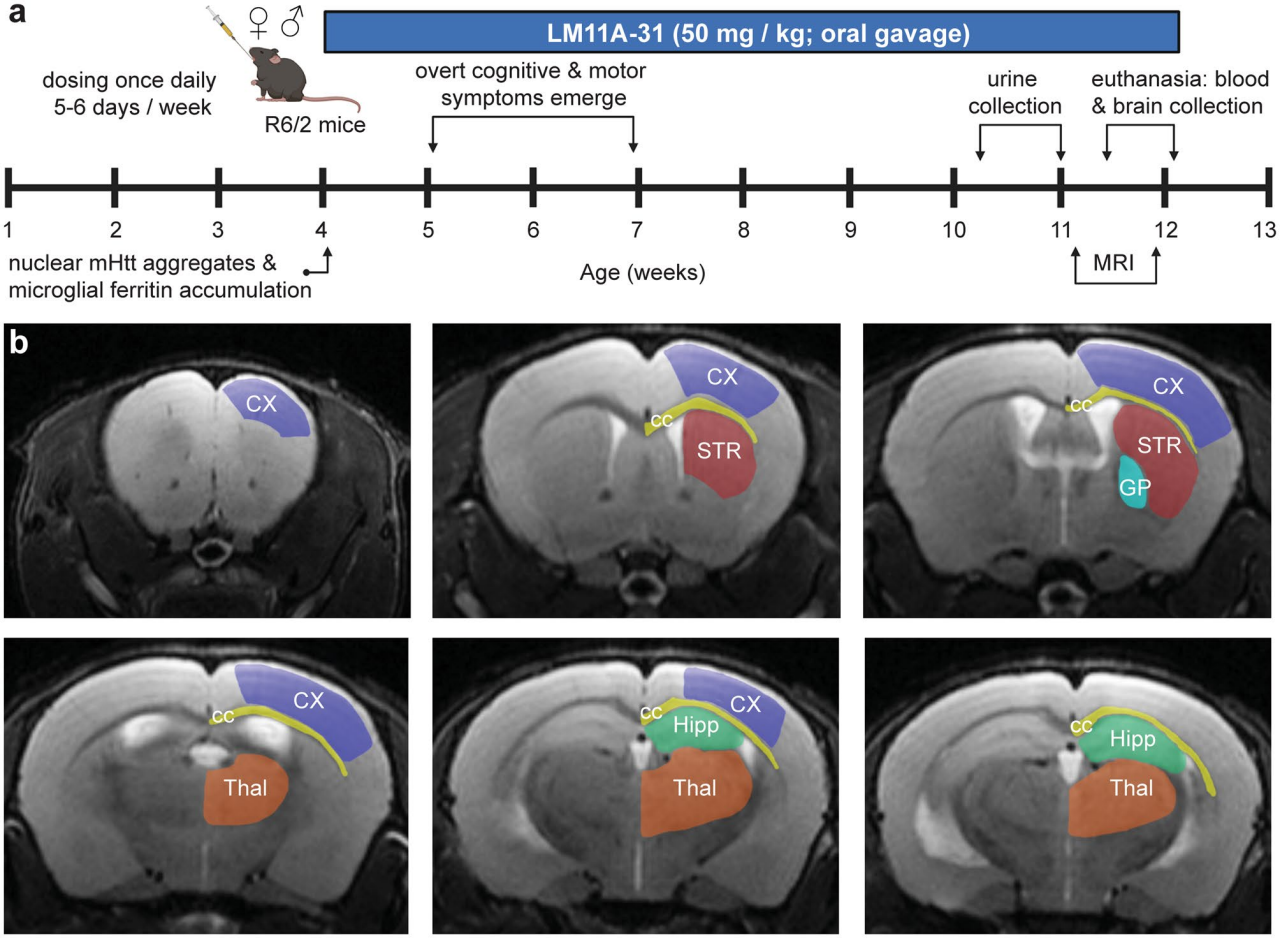
*Experimental timeline and brain regions of interest.***a** Experimental timeline showing that oral gavage dosing of male and female R6/2 mice and their wild-type (WT) littermates with LM11A-31 (50 mg/kg, once daily 5–6 days/week) started at 4 weeks of age until euthanasia ~ 7–8 weeks later. At 4 weeks, nuclear mutant huntingtin (mHtt) aggregates and microglial ferritin accumulation have been detected in R6/2 striatum and, at 5–7 weeks of age, motor and cognitive symptoms manifest. Age ranges at which urine, blood, and brain collection occurred and MRI was performed are noted. Created with BioRender.com. **b** T_2_-weighted MR images showing the delineation of the brain regions of interest (ROIs) used for analysis of volumetry, relaxometry, and DTI metrics. Representative MR images showing the anatomical localization of the following ROIs in one hemisphere: cortex (CX, purple), striatum (STR, red), corpus callosum with contiguous external capsule (CC, yellow), globus pallidus (GP, turquoise), dorsal hippocampus (Hipp, green), and rostral thalamus (Thal, orange). All measures were taken bilaterally.

This study was designed to identify non-invasive biomarkers to monitor the treatment response of potential HD therapeutics using as a prototype the small molecule p75^NTR^ ligand, LM11A-31, in the R6/2 mouse model of HD. R6/2 mice are transgenic for the 5′ end of the human HD gene carrying 100–190 glutamine (CAG) repeats [23] and are a good model of the anomalous splicing of mutant huntingtin that occurs in HD [24]. R6/2 mice are widely used for HD preclinical studies because they rapidly and reliably develop robust HD-related motor/cognitive deficits and pathology [23, 25]. They develop nuclear aggregates of mHtt and microglial ferritin accumulation as early as 4 weeks of age and progressive cognitive and motor deficits starting at 5–7 weeks [23, 25, 26]. These experiments utilized a 2 × 2 cross-sectional study design [wild-type (WT)/R6/2 × vehicle (Veh)/LM11A-31] with random group assignment. A cross-sectional study design was used since longitudinal studies of R6/2 mice are impractical, especially in those with shorter CAG repeats (< 130), given their severe phenotype, short lifespan, and proclivity to handling-induced seizures. Male and female R6/2 mice and their age-matched WT littermates were given LM11A-31 (50 mg/kg, P.O., once daily by oral gavage 5–6 days/week) for ~ 7–8 weeks starting at 4 weeks of age (*n* = 13–17 mice/group; *n* = 9–12 male and 4–5 female mice/group). MRIs were performed on a subset of these mice (*n* = 8–16 mice/group) as determined by scanner availability. The number of mice contributing to each analysis is provided in the figure captions. The group size needed to obtain statistical significance was determined based on previously published studies using these mice [8, 27]. As illustrated in the experimental timeline (Fig. 1a), urine was collected from mice at 10–11 weeks of age, neuroimaging studies were performed ~ 5–7 days later at 11–12 weeks of age, and plasma and brains were collected at the time of euthanasia (~ 2–5 days after MRIs). Quantitative neuroimaging and histological analyses were conducted by experimenters that were blind to treatment and genotype.

### Mice, Husbandry, and Genotyping

All animal procedures were conducted in accordance with the National Institutes of Health Guide for the Care and Use of Laboratory Animals using protocols approved by the Institutional Animal Care and Use Committee at Stanford University. These protocols included efforts to minimize animal suffering and the numbers used. Breeding pairs of R6/2 mice were purchased from Jackson Laboratories [female hemizygous ovarian transplant B6CBA-TgN (HD exon1)62; JAX stock #006494]. Males and females from litters born to these breeding pairs (R6/2 mice and WT littermates) were used in this study. Mice were group-housed in a pathogen-free animal facility with a 12-h light-dark cycle (on 6 am, off 6 pm); genotypes were mixed in a cage but genders were not. All mice received cotton nestlets and paper tubes with water and food freely available. Tail DNA was used for genotyping via real-time PCR by TransnetYX Inc. (Cordova, TN) and CAG repeat number measurement via ABI GeneMapper 4.0 by Laragen Inc. R6/2 mice in this study had an average of 128 ± 1.9 (mean ± SD) CAG repeats.

### Treatment with the p75^NTR^ Ligand LM11A-31

LM11A-31 is a small molecule p75^NTR^ ligand with a half-life in mouse brain of 3–4 h (single oral gavage dose of 50 mg/kg) [14, 28, 29]. Previous reports from our laboratory detail its chemical structure, pharmacokinetics, and pharmacodynamics [14, 29, 30]. LM11A-31 was custom-manufactured by Ricerca Biosciences at > 99% purity in a sulfate salt form (50 mg of salt contains 30 mg of the free base). It was dissolved in sterile water and given to experimental groups after 4 h of fasting at 50 mg/kg (10 ml/kg) via oral gavage once daily 5–6 days/week. Vehicle groups received water using the same paradigm. The LM11A-31 dose was chosen based on brain concentrations and biological effects determined in previous *in vivo* studies [14, 29]. This dose and dosing paradigm was used previously by our laboratory in multiple HD mouse models, including R6/2 mice, to reduce mHtt-related neuropathology and neuroinflammation and to improve motor and cognitive outcomes [8].

### Magnetic Resonance Image Acquisition and Post-acquisition Processing

*In vivo* MRI was performed using a horizontal bore Magnex Scientific scanner (Bruker Biospec) with 7.0 T field strength and Paravision v6.0.1 software at the Stanford University Small Animal Imaging Facility. To improve signal-to-noise ratio and resolution, a Bruker MRI CryoProbe was used as a receive coil, increasing the sensitivity of *in vivo* probes by a factor of 2.5–5 compared with room temperature coils by reducing the operating temperatures of the radiofrequency coil and preamplifier. Mice were anesthetized with isoflurane gas (2–3% induction, 1.5–2% maintenance with 40% O_2_), and a sterile lubricant was applied to their eyes to prevent drying. Mouse heads were immobilized in a plastic bed with a bite and ear bar and a nose cone for anesthesia delivery. Body temperature and respiration rate were monitored throughout the scan and appropriately adjusted (36–37 °C, 30–90 breaths per min). The head was positioned using a localizer scan. During a single scan session of ~ 40 min, images were acquired using three different sequences in the following order: T_2_-weighted fast spin-echo, diffusion-weighted multiple spin echo, and multi-echo gradient echo (MGE). Due to time limitations on MRI access, some mice (*n* = 1–3 mice per treatment group) only received T2 and/or MGE scans (for the exact number of mice per scan, see figure captions). Acquisition parameters are detailed below.

**T2-Weighted Images.** Images of coronal brain slices were obtained within a sample box positioned at the caudal end of olfactory bulbs spanning the forebrain and midbrain to the start of the cerebellum. Images were acquired over 7 min using T2-weighted fast spin-echo sequences with a repetition time (TR) of 4000 ms, an echo time (TE) of 58.5 ms, a field of view (FOV) of 20 × 20 mm^2^, a matrix size of 256 × 256, 36 slices of 0.3 mm thickness, and the number of excitations was 9. For volumetric analysis, a study-specific atlas was constructed by combining T_2_-weighted MR images from 10 WT and 10 R6/2 mice using the advanced normalization (ANTS) and multivariate template construction tools [31] (https://github.com/ANTsX/ANTs/blob/master/Scripts/antsMultivariateTemplateConstruction2.sh). Pre-defined regions of interest (ROIs) from both brain hemispheres were manually delineated on each slice of the T_2_-weighted images (orthogonal view) using an overlay in FSLView (v4.0.1) [32, 33]. The Franklin and Paxinos (2008) mouse brain atlas was used as a guide. ROIs included (Fig. 1b) striatum (dorsal, consisting of caudate and putamen) [Bregma (Bg) 1.54 mm to − 0.34 mm], globus pallidus [Bg − 0.22 mm to − 0.46 mm], cortex [including the frontal, motor (M1, M2), and somatosensory (S1) cortices; Bg 2.80 mm to − 1.70 mm], dorsal hippocampus [Bg − 1.06 mm to − 2.54 mm], rostral thalamus [Bg − 1.06 mm to − 2.54 mm], and corpus callosum + external capsule (ec) [1.70 mm to − 2.54 mm]. Right and left sides were traced separately except for midline structures (e.g., thalamus, corpus callosum). The ROIs were saved in Neuroimaging Informatics Technology Initiative (NIfTI) format, and the T_2_-weighted images from each scan were registered to the study-specific atlas using the ‘reg_aladin’ tool from the niftireg toolkit (http://cmictig.cs.ucl.ac.uk/wiki/index.php/NiftyReg). Transformations were applied to the manually traced study atlas ROIs resulting in individualized ROIs for each scan in subject space. These ROIs were visually inspected for each scan and manually edited to exclude spurious voxels by a blinded rater using FSLview. Volumetric data on the subject-specific ROIs and total brain volume were obtained via ITK-SNAP’s c3d command (http://www.itksnap.org/pmwiki/pmwiki.php?n=Convert3D.Documentation) [34]. Sex differences in regional brain volumes, including striatum, cortex, and/or hippocampus, were not evident in mid- to end-stage R6/2 mice or YAC128 mice [35–39]; thus, we pooled males and females for analysis of the T_2_-weighted images and subsequent measures. Similarly, hemispheric differences are infrequent with HD-related atrophy [40]; thus, right and left hemisphere ROI volumes were summed to compute the total ROI volume for each structure. In addition to absolute volumes, ROI volumes were divided by total brain volume to assess whether the volumes of individual brain structures were reduced relative to whole brain changes.

### Diffusion Tensor Imaging (DTI) and Neurite Orientation Dispersion and Density Imaging

For diffusivity metrics, a diffusion-weighted multiple spin-echo EPI sequence was used to acquire images (scan duration: 19 min 24 s) with the following parameters: TR = 400 ms, TE = 20.4 ms, one signal average, one repetition, 17 mm slice thickness, image size = 40 mm × 40 mm × 52 mm, FOV = 13 mm × 13 mm × 17 mm, 25 diffusion directions, 90° flip angle, and fat suppression on. Diffusion-weighted images were reconstructed from DICOM to NIfTI format with the ‘dcm2niix’ tool. DTI metrics were computed using FSL’s ‘dtifit’ tool (FSL v5.0.10)[32, 33]. For R2* relaxometry, eight T2*-weighted gradient-echo volumes (5 ms echo spacing) were acquired with a TR of 1456 ms, a TE of 3.5 ms (8 images with 5 ms spacing), two signal averages, one repetition, 50° flip angle, 0.3 mm slice thickness, and a FOV of 14 mm × 20 mm. R2* was computed by linear fitting after log transformation. All analyses were performed in subject space, and each voxel’s mean diffusivity (MD) and fractional anisotropy (FA) were averaged based on the same ROIs described above. NODDI metrics were calculated using the publicly available NODDI MatLab tool box (http://mig.cs.ucl.ac.uk/index.php?n=Tutorial.NODDImatlab). Single shell data allows for the fitting of orientation dispersion index (ODI), while two shells are required to fit neurite density measures [41]. With our one shell of DTI data, only ODI measurements were used. All data were calculated in subject space, and average ROI ODI was computed based on the individualized ROIs described above. Three mice (one R6/2-Veh, two R6/2-C31) were excluded from these analyses due to technical errors involving the diffusion acquisition.

### Urine Collection

Mice received a subcutaneous injection of 0.1 ml saline 1 h before urine collection, which occurred ~ 14–15 h after receiving their daily dose of LM11A-31. They were then scruffed and held over a disposable plastic container (a new container was used with each mouse). Gentle pressure was applied to the mouse’s lower back and belly to increase the imminence and the amount of urination. This process was repeated daily for up to 1 week to collect a sufficient volume of urine (~ 250 µl) to assay p75^NTR^-ecd levels. Voided urine was collected with a pipette and transferred to a centrifuge tube on ice. Urine was centrifuged (1000×*g*) for 10 min at 4 °C, and then, the supernatant was collected and stored at − 80 °C until assayed.

### Blood Collection and Brain Tissue Preparation

One-hour post-injection with LM11A-31 or vehicle, R6/2 and WT mice were deeply anesthetized with avertin and blood samples were drawn via cardiac puncture with a heparin-coated syringe and placed in EDTA-coated centrifuge tubes on ice. Between 30 and 45 min after collection, blood samples were centrifuged (1000×*g*, 10 min, 4 °C), and the supernatant was collected and designated plasma, which was stored at − 80 °C until use.

After blood samples were drawn, mice were immediately transcardially perfused with saline solution, and their brains were removed rapidly. The striatum was dissected from one brain hemisphere and flash frozen at − 80 °C until use for Western immunoblotting. The other brain hemisphere was immersion-fixed overnight in 4% paraformaldehyde in 0.1 M phosphate buffer (PB; pH 7.4), cryoprotected in 30% sucrose/PB, and sectioned (40 µm, coronal) using a freezing microtome for use in histological procedures.

### Quantification of p75^NTR^-ecd Levels in Urine

To measure p75^NTR^-ecd levels in urine, we developed an electrochemiluminescence (ECL)-based sandwich immunoassay using the Meso Scale Discovery (MSD, Rockville, MD) platform. Multi-Array® 96-well plates (MSD) with electrodes at the bottom of the wells were coated overnight with capture antibody (anti-p75^NTR^-ecd made in mouse; R & D Systems, cat # AF1157) diluted (1 µg/ml) in phosphate-buffered saline (PBS). Blocking was performed using 3% MSD Blocker A in PBS for 1 h. Blocking and all subsequent steps were performed on an orbital shaker (700 rpm) at room temperature (RT) unless otherwise noted, and incubations were followed by 3 washes with wash buffer (MSD). During blocking, samples were thawed at 37 °C and diluted 1:4 with Diluent 41 (MSD), ensuring the samples had a neutral pH (6.8–7.5) for the assay. Lyophilized recombinant mouse p75ecd-Fc standard (Biosensis, from ELISA kit cat # BEK2220) was reconstituted and diluted with Diluent 41. The standard (1 zero and 7 non-zero concentrations for standard curve) and samples from each treatment group were added to the plate (25 µl/well) in duplicate (per mouse) and incubated overnight at 4 °C (700 rpm). After 3 washes, the detection antibody (anti-p75^NTR^ecd made in rabbit; Advanced Targeting Systems, cat # AB-N01ap) was diluted to 1 µg/ml with 1% MSD Blocker A and added to the wells (25 µl/well) for 1 h. After washing, an MSD SULFO-TAG™ labeled anti-rabbit secondary antibody was diluted to 0.5 µg/ml with 1% MSD Blocker A and added to the wells (25 µl/well) for 1 h. Plates were washed, MSD Read buffer (diluted 1:2 in dH_2_O) was added to the wells, and the electrochemiluminescent (ECL) signal was detected with a QuickPlex SQ120 instrument (MSD). Discovery Workbench 4.0 software (MSD) was used to generate a calibration curve to which ECL signals were fit to determine analyte concentrations. To control for variability that may occur with total concentrations of urine sample, creatinine levels were measured from undiluted samples using a QuantiChrom™ Creatinine assay kit (BioAssay Systems, Hayward, CA), according to the manufacturer’s instructions. Results from duplicate samples were averaged per mouse and are expressed as ng p75^NTR^-ecd/mg creatinine.

### Quantification of Plasma Levels of Pro-inflammatory Cytokines

Levels of ten cytokines were measured in the plasma of mice from each treatment group using a V-PLEX Pro-inflammatory Panel 1 Mouse Kit (MSD), an ECL-based multiplex sandwich immunoassay. Diluent 41 (MSD) was used to dilute and/or reconstitute samples (1:2 dilution), calibrators (standard), and controls (mouse serum spiked with calibrators at three concentrations, as supplied with the kit) before adding to the plate in duplicate for incubation overnight at 4 °C (700 rpm). Procedures and analysis were performed according to the kit instructions and were similar to that described above for the p75^NTR^-ecd MSD assay.

### Western Immunoblotting

Brain tissue was prepared for Western blotting, as described previously [27]. Briefly, tissue homogenates were prepared in RIPA lysis buffer containing protease and phosphatase inhibitors. Protein samples from each genotype and treatment group were electrophoresed through a 26 well NuPAGE 4–12% Bis-Tris Gel with MOPS SDS running buffer (Invitrogen) and transferred to polyvinylidene difluoride membranes (Immobilon-FL, Millpore). Membranes were probed using the transferrin receptor antibody (1:1,000 ThermoFisher) and α-tubulin (Sigma) as a loading control. Secondary antibodies (ferritin: IRDye® 800CW, tubulin: IRDye® 680RD) were imaged with an Odyssey® CLx near-infrared fluorescence imaging system (Li-Cor Biosciences). Immunoreactive bands were manually outlined, and densities were measured using Image Studio Lite software (Li-Cor Biosciences). The densities of immunoreactive bands were expressed as a fraction of tubulin in the same lane. Samples were run in duplicate per mouse, and the data were normalized to the WT-Veh group of that gel then averaged.

### Nissl Staining, Immunohistochemistry, and Image Analysis

For Nissl staining, every 8th coronal fixed-brain section (40 µm) was mounted onto slides and stained with 0.5% Cresyl violet in distilled water. Volumes of the striatum, cortex (motor and somatosensory), dorsal hippocampus, and corpus callosum (including the contiguous external capsule) were estimated in one hemisphere using unbiased stereology via the Cavalieri method within StereoInvestigator v11.07 (MBF). ROIs were delineated using the criteria described above and shown in Fig. 1b.

For immunostaining, free-floating sections were processed for localization of the iron storage protein ferritin (1:1000; Proteintech) using procedures described previously [26]. Images of ferritin immunostaining were acquired from 2 to 3 striatal sections per mouse [2 sample fields/striatum] using the quick-full focus option (through 25 μm of tissue in the Z-plane) of a Keyence BZ-9000 microscope (20× objective). The images were maximally projected and analyzed with ImageJ using the subtract background and auto-threshold commands. Data are presented as the mean percent area occupied by ferritin immunostaining per section/mouse. Immunostaining was performed in multiple sets so quantifications were normalized to the WT-Veh group of that staining set. All figures were created in Adobe Illustrator CS6 v.16.

### Statistical Analysis

GraphPad Prism (version 8) software was used for all statistical analyses. Data normality was assessed using the Kolmogorov-Smirnov test. Statistical significance of mean differences between normally distributed continuous variables with equal variances was tested by one-way analysis of variance (ANOVA) with a Fisher’s LSD post hoc test with planned comparisons. Significant differences between non-normally distributed variables were assessed using the non-parametric Mann-Whitney test and those with unequal variances the Welch’s *t* test. Multiple comparisons were addressed for each MRI metric and the plasma cytokine analysis using the Benjamini, Krieger, and Yekutieli method [42], with the false discovery rate at 5%. Pearson correlation coefficients (*r*) were used to test for associations. Values that were two standard deviations from the mean (criteria determined a priori) were removed as statistical outliers (as noted in the figure captions when necessary). Results are expressed as group mean ± standard error of the mean (s.e.m.), and statistical significance was set at p ≤ 0.05. Effect size (Cohen’s d) and power (determined post hoc; *α* = 0.05) were computed with G*Power (v.3.1.9.4) software (Table 1). Cohen’s *d* is the effect size allowing the standardized differences between groups to be compared. The number of mice and the statistical test(s) used for each analysis is specified in the figure captions.

### Machine Learning Analyses

Machine learning models, including logistic regression, feature selection, and classification, were implemented using pre-processed standardized data (Z-scores) and scikit-learn 0.23.2 in Python [43]. Logistic regression is a simple machine learning algorithm used to analyze multiple explanatory variables and to determine the magnitude of the association between predictor and response variables [44]. The 27 features (neuroimaging or biofluid markers) that showed significant effects in standard statistical analyses were used as predictor variables. Logistic regression coefficients were computed to compare genotype (WT-Veh versus R6/2-Veh) or treatment (R6/2-Veh versus R6/2-C31; *n* = 12–13 mice per group for each comparison).

A more complex two-step machine learning analysis was also performed using feature importance ranking by one of two algorithms, support vector machine (SVM) with a linear kernel or extreme gradient boosting (XGB; v1.3.0), followed by classification with recursive feature elimination using either k-nearest neighbors (KNN; *k* = 3) with distance-type weighting or random forest classifier (RFC) with 200 estimators [45–48]. The four models (SVM-KNN, SVM-RFC, XGB-KNN, or XGB-RFC) were run for iteratively decreasing the number of features, from all 27 to 2, to distinguish features that classify subjects by genotype or treatment. For these analyses, a subset of the data (training set) is used to create an algorithm that uses the remaining data (test set) to assess the model’s ability to classify new data. The data was randomly divided in about half for the training-test split (*n* = 6 mice/group for the test set, *n* = 6–7 mice/group for training) since the number of samples was low compared with features. Given the limited number of animals, 1000 permutations of randomly selected training and tests sets were run for each model setup to promote robustness and reliability. Model performance was compared using the mean values of prediction accuracy, precision, and recall.

## Results

### LM11A-31 Alleviates R6/2 Brain Atrophy as Assessed with *In Vivo* Volumetric MRI: Histological Confirmation with *Ex Vivo* Stereology

**Fig. 2:**
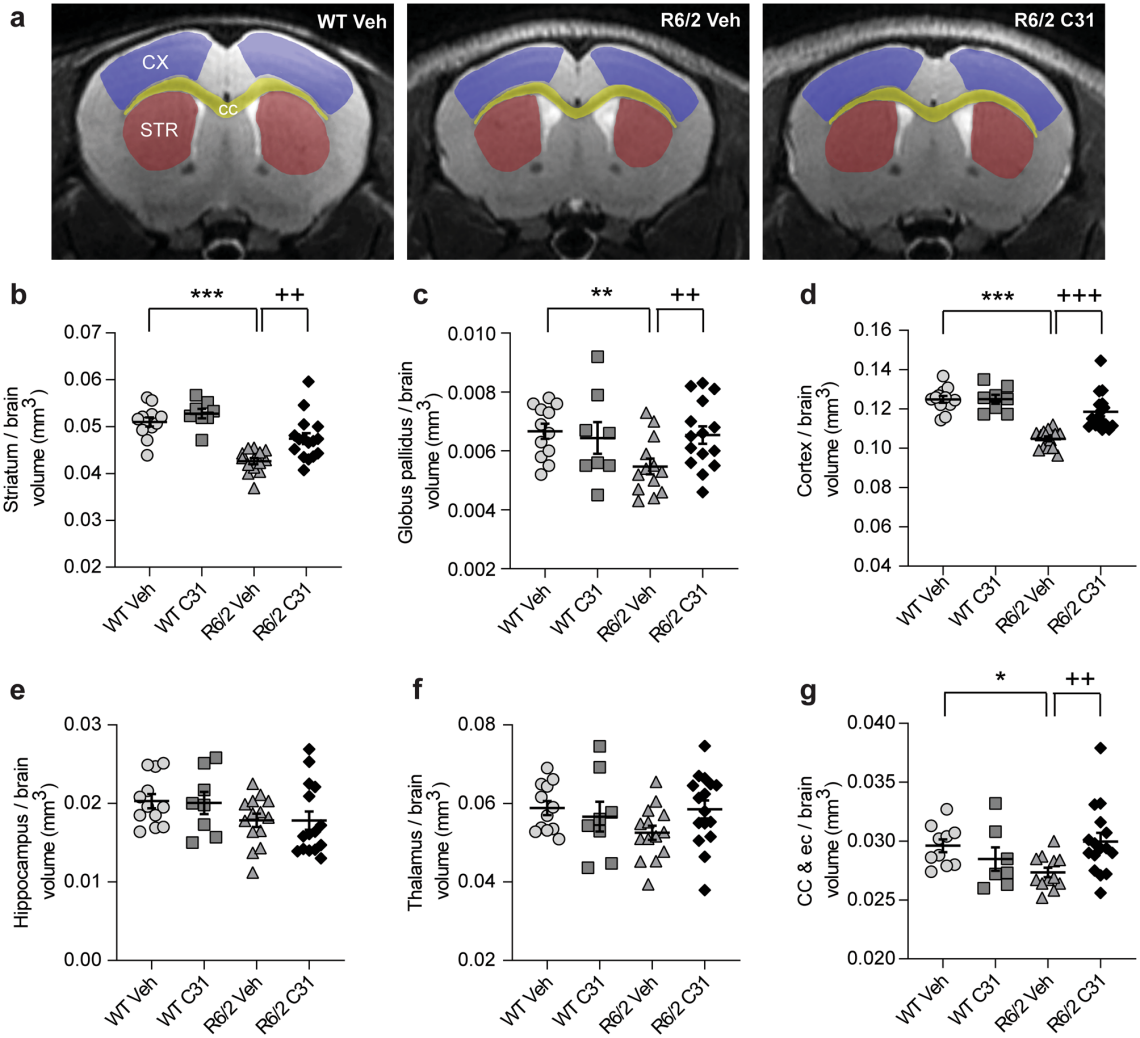
*LM11A-31 alleviates regional volume reductions, as assessed with MRI, in R6/2 mice.***a** Representative T_2_-weighted MR images of a coronal section from a WT and R6/2 mice given vehicle (Veh) and an R6/2 mouse given LM11A-31 (C31) at 11–12 weeks of age. Shown are three of the regions of interest (ROIs) that exhibited significant volume reductions: striatum (STR, red), cortex (CX, purple), and corpus callosum (cc, yellow)/contiguous external capsule (ec). Quantification of the volumes of the **b** striatum, **c** globus pallidus, **d** cortex, **e** dorsal hippocampus, **f** thalamus, and **g** corpus callosum as measured from T_2_-weighted MR images and adjusted for total brain volume. For absolute volumes (*i.e.*, not adjusted for total brain size), see Suppl. Fig. 1. The striatum, globus pallidus, cortex, and corpus callosum/ec were significantly smaller in R6/2-Veh compared with WT-Veh mice. C31 alleviated these decreases. WT-Veh *n* = 12 mice; WT-C31 *n* = 8; R6/2-Veh *n* = 14; R6/2-C31 *n* = 16. Results are expressed as mean ± s.e.m. Statistical significance was determined with an ANOVA and Fisher’s LSD with an FDR adjustment. *p = 0.025, **p ≤ 0.01, and ***p ≤ 0.0005 versus WT-Veh; ^++^*p* = 0.01 and ^+++^*p* = 0.0005 versus R6/2-Veh.

Previous *in vivo* volumetric MRI studies have shown decreased regional brain volumes in HD mouse models, including R6/2 mice [35, 38, 39, 49]. Here, we investigated whether such reductions could be alleviated with LM11A-31 and if this treatment effect could be detected with MRI volumetry in R6/2 mice at 11–12 weeks of age. The absolute volumes of each of the ROIs examined were significantly smaller in vehicle-treated R6/2 mice than in WTs, as was the total brain volume (Suppl. Fig. 1). Given the reduced total brain size, we also examined whether the volumes of individual brain structures were decreased relative to whole brain changes to assess regional rather than global atrophy [50]. After normalizing for total brain size, volumes of the striatum and cortex were both reduced by 16 ± 1% (mean ± s.e.m.; Cohen’s *d* = 3.13 and 3.66, respectively; see Table 1 for effect sizes) and the globus pallidus by 22 ± 1% (Cohen’s *d* = 2.00) in vehicle-treated R6/2 mice relative to WTs (Fig. 2a–d). The whole-brain adjusted volumes of the dorsal hippocampus and rostral thalamus were not statistically different from WTs (Fig. 2e, f). White matter atrophy was also detectable as evidenced by a 9 ± 1% (mean ± s.e.m.; Cohen’s *d* = 1.90) reduction in proportionalized volume of the corpus callosum and contiguous external capsule (ec) in vehicle-treated R6/2 mice (Fig. 2g). LM11A-31 did not affect total brain size (Suppl. Fig. 1g) or ROI volume in WT mice. However, LM11A-31 did alleviate volume reductions in the R6/2 striatum, globus pallidus, cortex, and corpus callosum/ec with 10 ± 3 to 30 ± 6% larger volumes compared with R6/2-Veh mice (Fig. 2) (Cohen’s *d* = 0.97–2.00; Table 1).

**Fig. 3:**
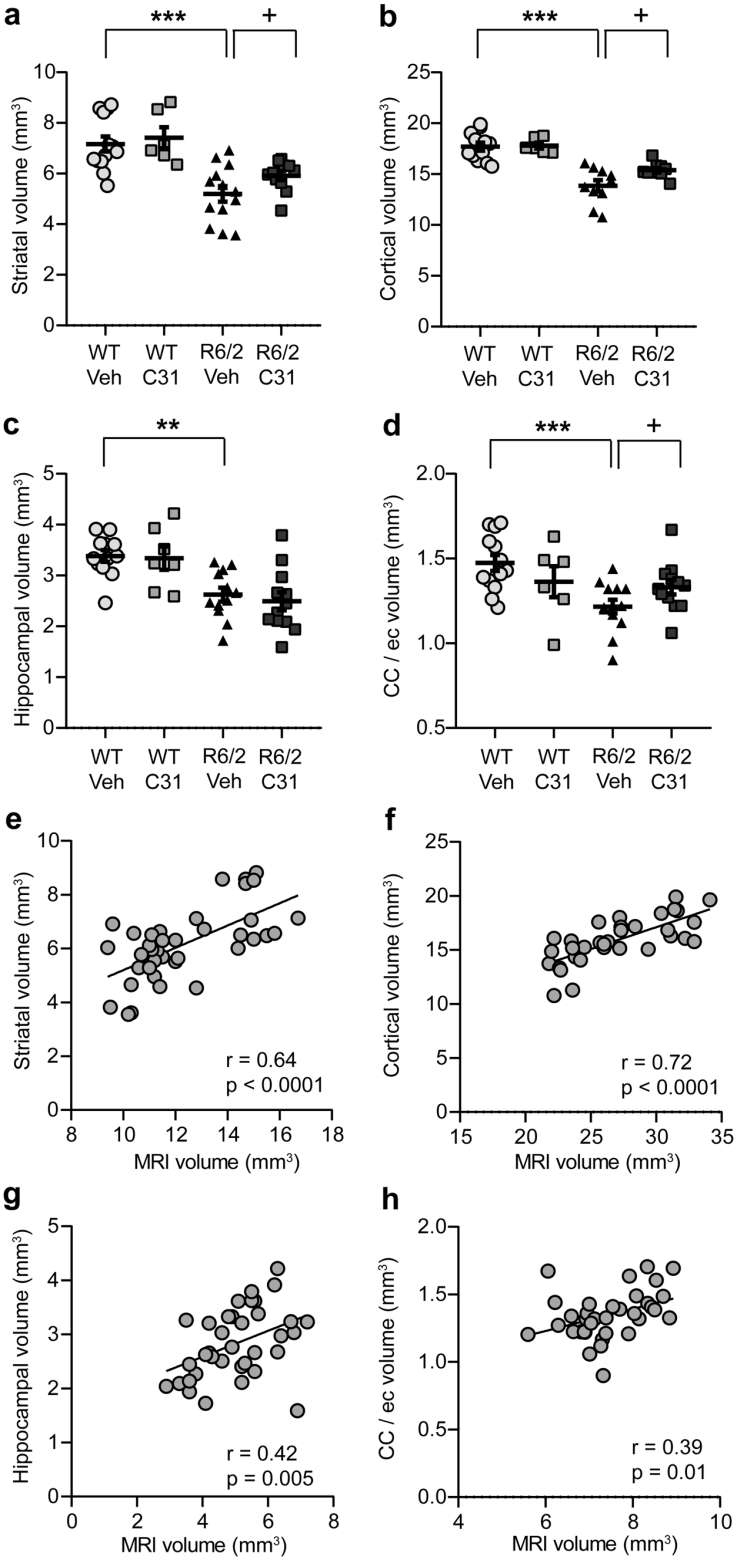
*Regional volume reductions, as assessed ex vivo using Nissl-stained sections, correlate with those obtained with in vivo MRI.***a**–**d** Using unbiased stereology on Nissl-stained tissue from the MR imaged mice, the Cavalieri-estimated volumes of the **a** striatum, **b** cortex (including both the somatosensory and motor cortices), **c** dorsal hippocampus, and **d** corpus callosum (CC) including the contiguous external capsule (ec) were reduced in R6/2-Vehicle (Veh) mice compared with WTs. *n* = 6–13 mice/group. Results are expressed as mean ± s.e.m. Statistical significance was determined with an ANOVA and Fisher’s LSD or Welch’s *t* test for unequal variance. **p ≤ 0.001 and ***p ≤ 0.0001 versus WT-Veh; ^+^*p* ≤ 0.05 versus R6/2-Veh (Welch’s *t* test for striatum and CC comparisons). **e**–**h** Scatterplots with linear regression lines show that the absolute (i.e., not adjusted for total brain volume) volumes (mm^3^) of the **e** striatum, **f** cortex, **g** hippocampus, and **h** CC/ec quantified from T_2_-weighted MR images (*x*-axis) correlated with Cavalieri-estimated volumes (mm^3^) from Nissl-stained sections (*y*-axis). The experimental groups are combined for the analysis (*n* = 6–13 mice/group). Pearson correlation coefficients (*r*) and *p* values are shown.

Stereology was performed on Nissl-stained sections from MR-imaged mice to provide histological corroboration of volume reductions of four ROIs that were evaluated with structural MRI. Cavalieri-estimated volumes were reduced in R6/2-Veh mice by 27.4 ± 4.3% in the striatum (mean ± s.e.m.), 22 ± 3% in cortex (including both the somatosensory and motor cortices), 22 ± 4% in dorsal hippocampus, and 18 ± 3% in the corpus callosum/ec compared with WTs (Fig. 3a–d). The magnitude of these volume reductions was similar to those obtained after manual segmentation of ROIs on T_2_-weighted MR images (Fig. 2). Likewise, LM11A-31’s ameliorative effect on volume decreases was detected on the Nissl-stained sections, again with similar percent changes as seen with MRI. The *in vivo* absolute volumes of ROIs assessed via MRI correlated strongly with *ex vivo* volumes assessed using stereology on Nissl-stained sections (Fig. 3e–h). In all, *in vivo* MRI has sufficient sensitivity to detect disease-related structural abnormalities as well as LM11A-31 treatment effects in both gray and white matter of R6/2 mice.

### LM11A-31 Preserves Microstructural Integrity of Selective R6/2 Brain Areas

Since LM11A-31’s ameliorative effects on regional brain atrophy in R6/2 mice could be detected with MRI, we investigated if the ligand could also affect HD-related microstructural changes and disrupted connections, which often occur earlier in the disease course, as assessed *in vivo* using DTI [18]. DTI allows measurements of molecular diffusion properties of water, which can be affected by numerous factors, including the density and composition of neurons and their fibers, myelination, as well as intra-/extracellular volume and content [17, 18]. Two of the most commonly reported diffusion parameters are mean diffusivity (MD), which is the molecular diffusion rate, and fractional anisotropy (FA), which is the preferred direction of diffusion [17, 18]. Most DTI studies of pre-symptomatic HD gene carriers (HDGCs) and symptomatic HD patients revealed increased MD and decreased FA in white matter regions including the corpus callosum, while both MD and FA are increased in gray matter including the caudate, putamen, and globus pallidum [17, 18, 51–55]. Here, we examined the effects of LM11A-31 on the integrity of white matter tracts and the microstructure of gray matter using *in vivo* DTI to measure MD and FA in the brains of 11–12 week-old R6/2 mice and age-matched WTs with and without LM11A-31 treatment.

**Fig. 4:**
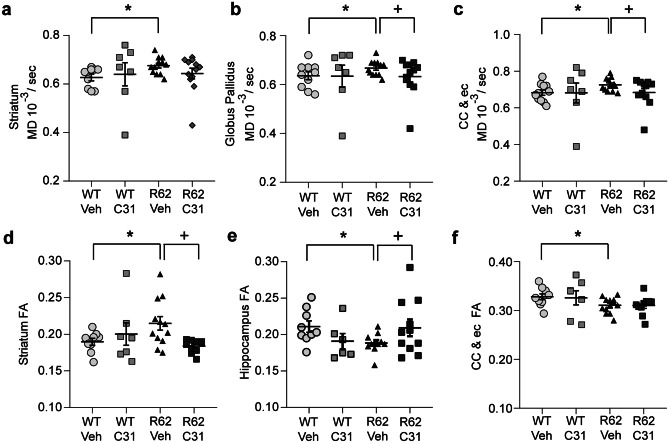
*LM11A-31 normalizes diffusivity metrics in subcortical regions of R6/2 mice.***a**–**c** Mean diffusivity (MD) and **d**–**f** fractional anisotropy (FA) values in the **a**, **d** striatum, **b** globus pallidus, **e** dorsal hippocampus, and **c**, **f** corpus callosum (cc)/contiguous external capsule (ec) of 11–12 week-old WT and R6/2 mice with and without LM11A-31 (C31) treatment. WT-Veh *n* = 10 mice; WT-C31 *n* = 7; R6/2-Veh *n* = 12; R6/2-C31 *n* = 10. Results are expressed as mean ± s.e.m. Statistical significance was determined with an ANOVA and Fisher’s LSD with an FDR adjustment. *p ≤ 0.05 versus WT-Veh; ^+^*p* ≤ 0.05 versus R6/2-Veh.

R6/2-Veh mice had significantly higher MD values compared with WTs in the striatum (Fig. 4a), globus pallidus (Fig. 4b), thalamus (Suppl. Fig. 2), and corpus callosum/ec (Fig. 4c). MD values did not differ between these groups in the cortex or hippocampus (Suppl. Fig. 2). LM11A-31 prevented these changes in the globus pallidus and corpus callosum/ec as mean MD values were significantly lower in the R6/2 mice given LMA11-31 versus vehicle and did not differ from WTs (Fig. 4b, c). LM11A-31 did not significantly affect MD values in WT mice or any of the other brain regions examined in R6/2 mice. Regarding FA values, vehicle-treated R6/2 mice had patterns of FA changes that were similar to those seen in studies of HD patients and other mouse models [17, 18]. FA significantly increased in the striatum (Fig. 4d) of R6/2-Veh mice but decreased in the hippocampus (Fig. 4e), corpus callosum/ec (Fig. 4f), cortex (Suppl. Fig. 2), and thalamus (Suppl. Fig. 2) compared with WTs; it was unaltered in the globus pallidus (Suppl. Fig. 2). LM11A-31 normalized FA values of R6/2 mice in the striatum (Fig. 4d) and hippocampus (Fig. 4e), but not the corpus callosum/ec (Fig. 4f), cortex, or thalamus (Suppl. Fig. 2). In all, LM11A-31 normalized certain diffusivity metrics in subcortical regions of R6/2 mice with the largest treatment effects concerning FA in the striatum (Cohen’s *d* = 1.32; Table 1).

### Effects of LM11A-31 on Neurite Orientation Dispersion

**Fig. 5:**
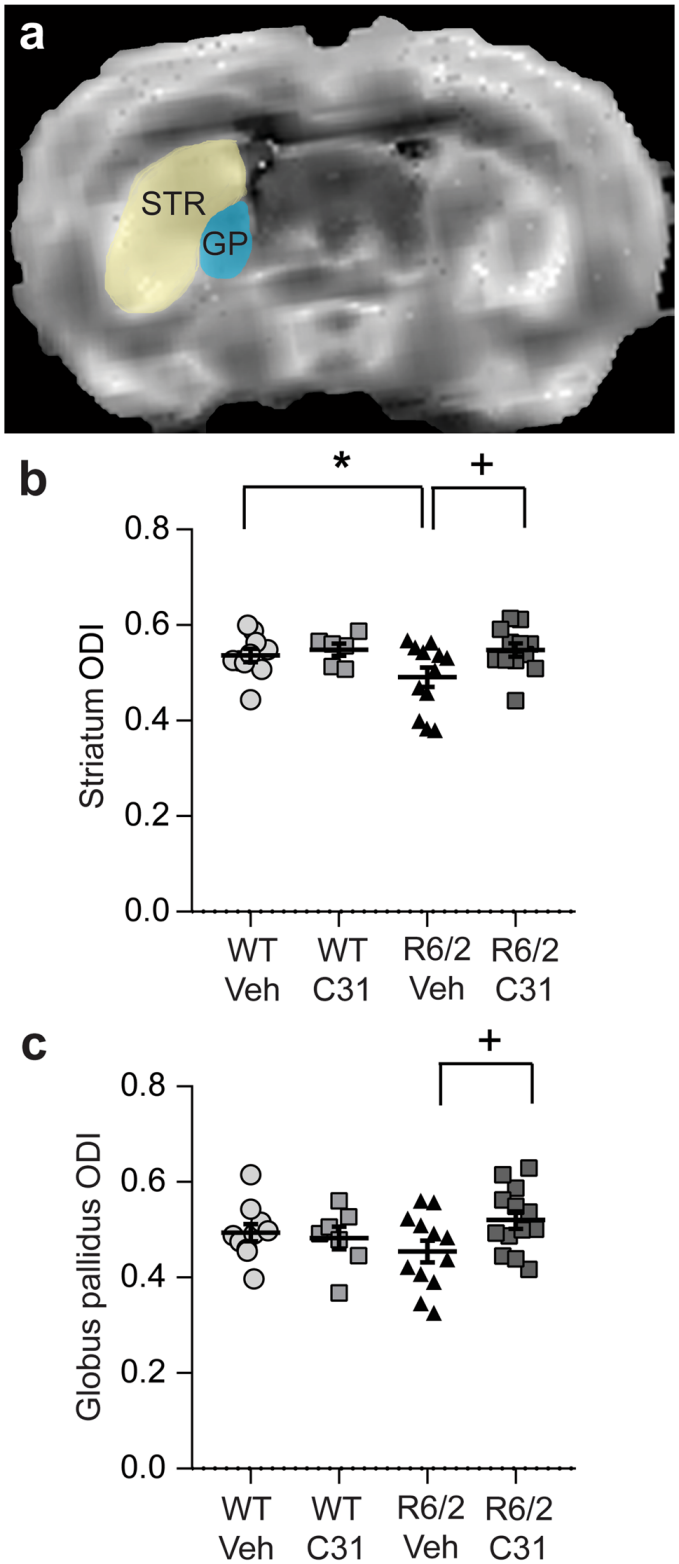
*Effects of LM11A-31 on the orientation dispersion index (ODI) in the striatum and globus pallidus of R6/2 mice.***a** Representative diffusion-weighted image of a coronal section of the striatum (STR) and globus pallidus (GP) from an WT-Veh mouse at 11–12 weeks of age. Quantification of the ODI in the **b** striatum and **c** globus pallidus of WT and R6/2 mice given vehicle (Veh) or LM11A-31 (C31). *n* = 7–12 mice/group. Results are expressed as mean ± s.e.m. Statistical significance was determined with an ANOVA and Fisher’s LSD. *p = 0.04 versus WT-Veh; ^+^*p* = 0.02 versus R6/2-Veh.

Diffusion MRI metrics, such as FA and MD, are useful as surrogate measures of microstructural tissue damage; however, alternative measures are needed to examine neurite loss and alterations to the arrangement of fibers. The NODDI technique was developed to more specifically characterize axonal pathology and gray matter changes with MRI [41]. One study of HD patients used NODDI metrics to show axonal density and organization abnormalities in premanifest HDGCs, including a decreased ODI in the white matter encompassing the basal ganglia [52]. We used the NODDI model to calculate the neurite ODI, which reflects neurite spatial configuration and can measure diffusivity in the extra-neurite compartment [41, 56]. R6/2 mice given vehicle had lower striatal ODI values than WTs and showed a non-significant decrease in the globus pallidus. In both brain regions, LM11A-31 prevented the decrease in ODI (Fig. 5). ODI values in the other ROIs did not differ significantly between genotypes (Suppl. Fig. 3).

### LM11A-31 Normalizes MRI Relaxometry and Iron Regulatory Protein Levels in R6/2 Striatum

**Fig. 6:**
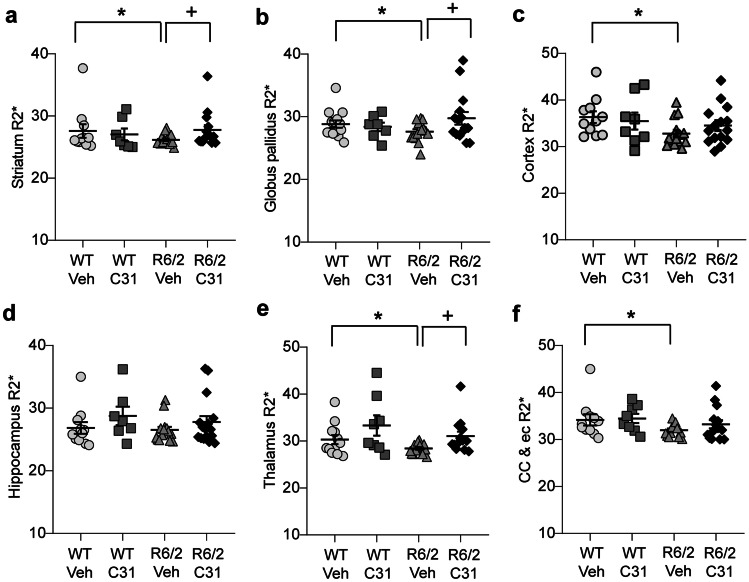
LM11A-31 normalizes mean relaxation rates (R2*) without ROI volume correction in WT and R6/2 mice.**a**–**f** R2* values without regional volume correction in the **a** striatum, **b** globus pallidus, **c** cortex, **d** hippocampus, **e** thalamus, and **f** corpus callosum (cc)/contiguous external capsule (ec) of WT and R6/2 mice given vehicle (Veh) or LM11A-31 (C31). WT-Veh *n* = 12 mice; WT-C31 *n* = 7; R6/2-Veh *n* = 14; R6/2-C31 *n* = 15. Results are expressed as mean ± s.e.m. Statistical significance was determined with an ANOVA and Fisher’s LSD or *t* test with an FDR correction. *p ≤ 0.05 versus WT-Veh; ^+^*p* ≤ 0.05 versus R6/2-Veh.

Many iron sensitive neuroimaging studies of HD patients indicate that iron levels increase in the striatum and globus pallidus and can be detected at pre-symptomatic disease stages [55, 57–65]. Various MRI techniques, including relaxation time, can measure local changes in magnetic susceptibility, which are often caused by iron bound to ferritin, an iron storage protein [64, 66]. Transverse relaxation rate (R2*) is one such technique that strongly correlates with chemically assessed iron concentrations with high R2* values associated with increased iron content [67, 68]. A study in late-stage R6/2 mice showed a decrease in T2* relaxation time (inverse of R2*) [38], which is consistent with a previous report of increased ferric iron and/or ferritin, particularly in dystrophic microglia, in striatum of R6/2 mice and early manifest HD patients [26]. Given this increase in ferritin-containing microglia and LM11A-31’s reductive effect on microglial activation in R6/2 mice [8, 26], we measured mean R2* in WT and R6/2 mice treated with LM11A-31 or vehicle predicting R2* increases indicative of increased ferritin levels. Unexpectedly, small but significant decreases in R2* values were seen in the striatum (Cohen’s *d* = 2.57), globus pallidus (Cohen’s *d* = 1.6), cortex (Cohen’s *d* = 1.83), thalamus, and corpus callosum/ec of R6/2-Veh mice compared with WTs (Fig. 6, Table 1). R6/2 mice treated with LM11A-31 did not differ from WTs and had higher R2* values than R6/2-Veh mice in the striatum, globus pallidus, and thalamus (Fig. 6).

Increased iron content can account for striatal shrinkage which can create bias in regional segmentation and spatial variations in relaxometry [69–72]. Moreover, R2* values are affected by heterogeneous iron distribution and depth gradients of iron concentrations exist [73, 74]. Thus, we performed an ROI volume correction of the R2* values, as done previously, to calculate iron accumulation per ROI volume [57]. After adjusting for ROI volume, R2* values were increased, which is the predicted direction for elevated iron, in each brain area examined in R6/2-Veh mice compared with WTs (Suppl. Fig. 4). These elevations were partially prevented by LM11A-31 only in the striatum of R6/2 mice (Suppl. Fig. 4). R2* values did not correlate with the volume of the ROIs examined except for the cortex and corpus callosum (Suppl. Fig. 5), similar to findings in HD patients using other MR-based relaxometry techniques [75, 76]. Thus, iron deposition per volume is increased in all brain areas examined in R6/2-Veh mice, and LM11A-31 prevents this elevation in the R6/2 striatum.

**Fig. 7:**
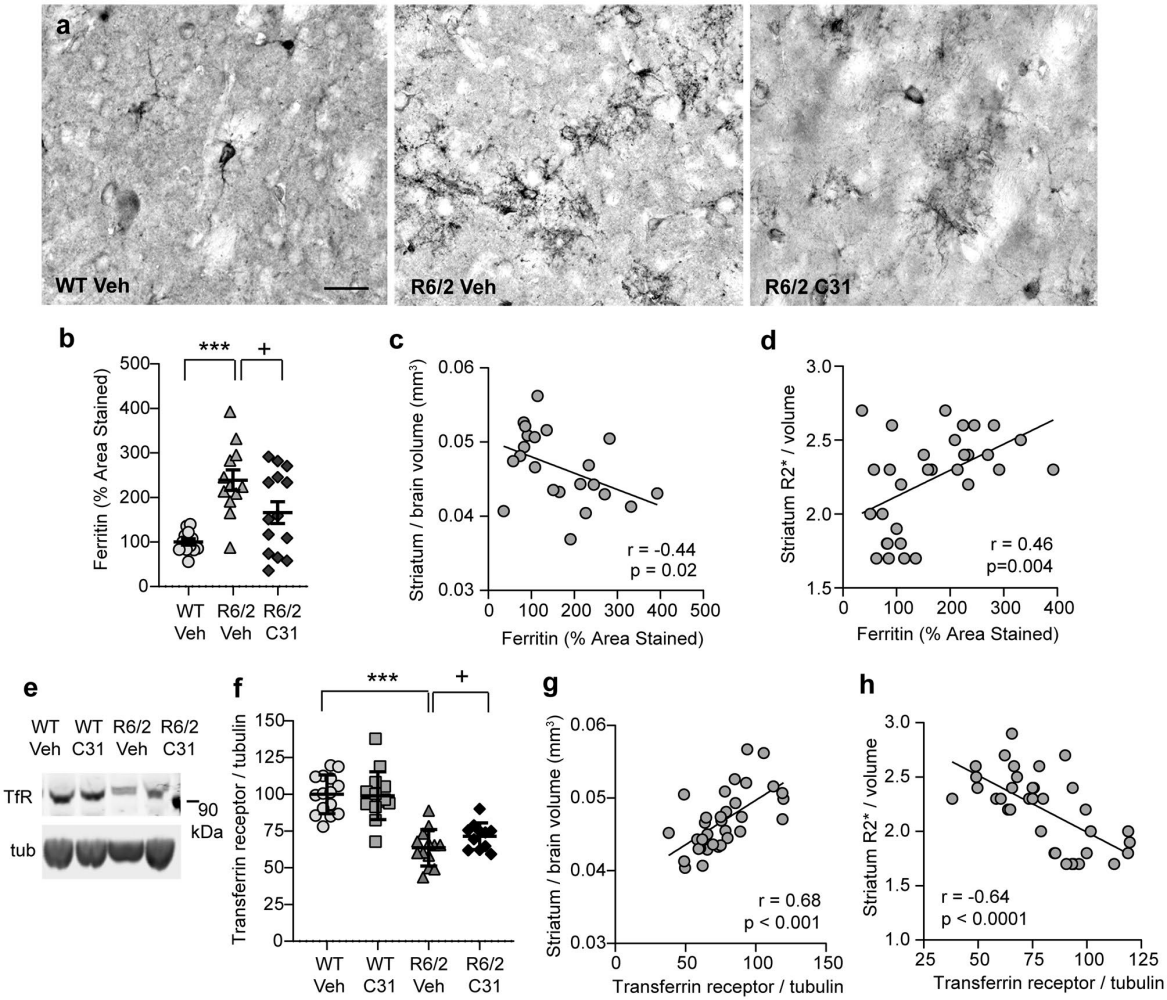
Iron regulatory protein levels are normalized in striatum of R6/2 mice treated with LM11A-31 and strongly correlate with mean relaxation rates (R2*) per volume.**a** Representative photomicrographs of ferritin immunostaining in the striatum of a WT-Veh mouse (*left*) and R6/2 mice given Veh (*middle*) or LM11A-31 (*right*) of MR imaged mice. Scale bar = 25 µm. **b** Quantification of the area occupied by ferritin immunostaining. *n* = 12–14 mice/group. Results are expressed as mean ± s.e.m. Statistical significance was determined with an ANOVA and Fisher’s LSD. ***p < 0.0001 versus WT-Veh; ^+^*p* = 0.02 versus R6/2-Veh. **c**, **d** The area of ferritin immunostaining negatively correlated with striatal volume adjusted for total brain volume (**c**) and positively correlated with R2* values adjusted for striatal volume (**d**). **e** Representative lanes from western immunoblots of striatal homogenates from MR imaged mice probed for transferrin receptor (TfR) and tubulin (tub) are shown. **f** Corresponding densitometric analysis of immunoblots. Means (± s.e.m.) were from 2 replicated runs for each mouse sample (*n* = 11–15 mice/group) and were normalized to the WT-Veh group run on the same gel. Statistical significance was determined with an ANOVA and Fisher’s LSD (***p < 0.0001 versus WT-Veh) and a *t* test (^+^*p* = 0.048 versus R6/2-Veh). **g**, **h** Transferrin receptor levels positively correlate with striatal volume/brain (**g**) and negatively correlate R2* values/striatum volume (**h**). The experimental groups are combined for the analysis. Pearson correlation coefficients (*r*) and *p* values are shown.

Since magnetic susceptibility is mainly due to ferritin-bound iron in gray matter [67, 68], we investigated whether the R2* changes seen here are associated with ferritin levels in the R6/2 striatum. As shown previously [26], the area of ferritin-immunostained cells resembling microglia and their processes more than doubled in the striatum of R6/2-Veh mice compared with WTs (Fig. 7a, b). Treating R6/2 mice with LM11A-31 alleviated this increase (Fig. 7b). Notably, the area of ferritin immunostaining negatively correlated with striatal volume and positively correlated with R2* values per volume (Fig. 7c, d). Another iron regulatory protein altered in HD brains is the transferrin receptor, which is involved in iron uptake into the cell. Transferrin is down-regulated in the presence of excessive iron as a protective mechanism against an overload of intracellular iron, and its levels are decreased in the R6/2 striatum [77, 78]. In this study, striatal levels of transferrin receptor were reduced by 36 ± 12% (mean ± SD) in R6/2-Veh mice compared with WTs (Fig. 7e, f). LM11A-31 slightly increased transferrin receptor levels in R6/2 striatum. Striatal transferrin receptor levels positively correlated with striatal volume and negatively correlated with R2* values/volume (Fig. 7g, h).

### LM11A-31 Reduces Plasma Levels of Certain Pro-inflammatory Cytokines

**Fig. 8:**
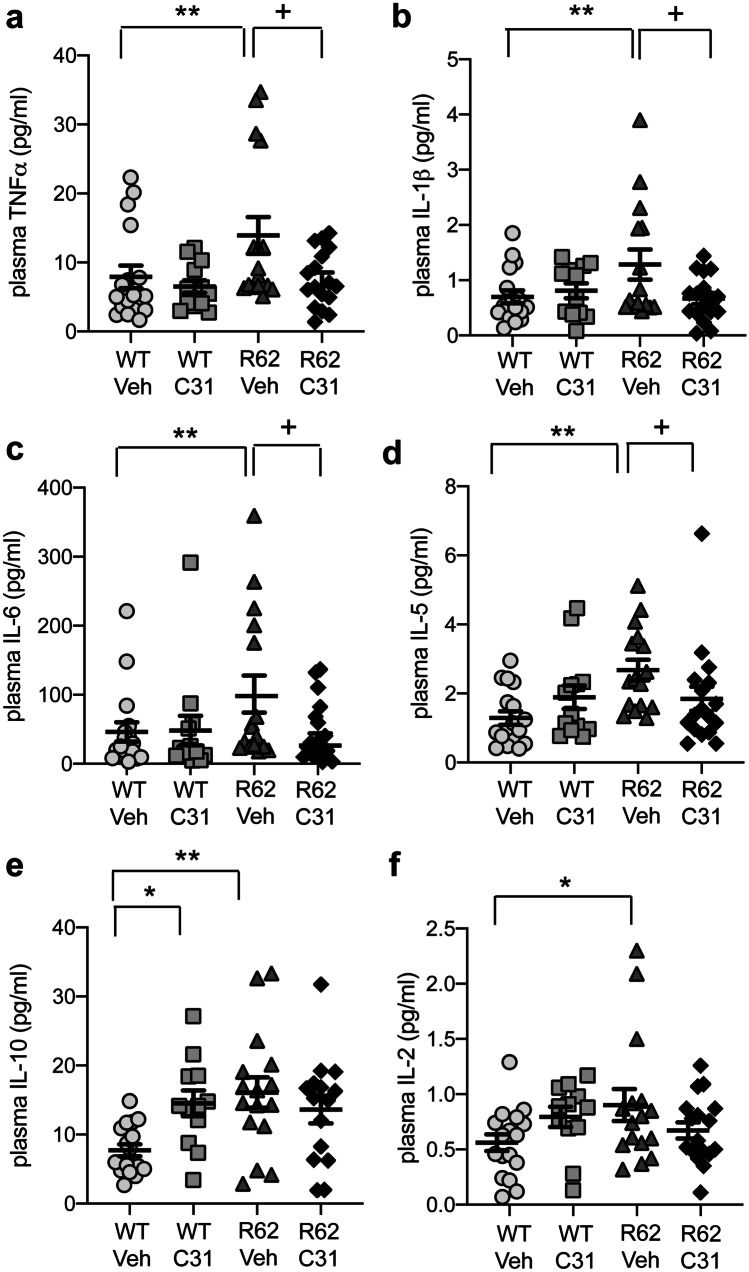
*Increases in plasma levels of certain cytokines are prevented in LM11A-31-treated R6/2 mice.***a**–**f** Levels of TNFα (**a**), IL-1β (**b**), IL-6 (**c**), IL-5 (**d**), IL-10 (**e**), and IL-2 (**f**) were increased in the plasma of R6/2 mice given vehicle (Veh) compared with WT-Veh mice. LM11A-31 alleviated increased levels of the former four cytokines. WT-Veh *n* = 17 mice; WT-C31 *n* = 13; R6/2-Veh *n* = 16; R6/2-C31 *n* = 17. Results are expressed as mean ± s.e.m. Statistical significance was determined with an ANOVA and Fisher’s LSD for IL-5 and IL-10. Concentrations of the other four cytokines in the WT-Veh and/or R6/2-Veh groups did not pass the Kolmogorov-Smirnov test for normality; thus, the non-parametric Mann-Whitney test was used to test for significant differences. FDR was applied to account for multiple comparisons. One R6/2-Veh mouse in the IL-1β analysis and one R6/2-C31 mouse from the IL-10 analysis were removed as statistical outliers. Detectable levels of IL-1β were below the assay limits for a WT-C31 and an R6/2-C31 mouse and for IL-10 in an R6/2-C31 mouse. *p ≤ 0.05 versus WT-Veh; ^+^*p* ≤ 0.05 versus R6/2-Veh.

Cytokines are released from activated microglia to mediate inflammatory responses, and their levels are elevated in the brains and/or plasma of HD patients and mouse models [79–85]. We previously showed that LM11A-31 decreased microglial activation in multiple HD mouse models and altered the functional phenotypes of microglia by reducing the concentrations of several cytokines that were elevated in the R6/2 striatum, including interleukin (IL)-6 and tumor necrosis factor (TNF)-α [8, 16]. Here, we investigated whether plasma levels of cytokines are also affected by LM11A-31. Levels of interferon-γ, IL-4, and IL-12p70 were near or below the detectable limit of the assay in many of the plasma samples (*n* = 4–10 mice in one or more groups); thus, data for these cytokines are not presented. Plasma levels of TNFα, IL-1β, IL-6, IL-5, IL-10, and IL-2 were significantly elevated in R6/2 mice given vehicle compared with WT mice (Fig. 8), while KC/GRO was unaltered (data not shown). The increase in plasma levels of four of these cytokines (TNFα, IL-1β, IL-6, and IL-5) was prevented in R6/2 mice treated with LM11A-31 (Fig. 8a–d). WT mice treated with LM11A-31 had elevated plasma levels of IL-10 compared with WT-Veh mice.

### Urinary p75^NTR^-ecd Levels Are Elevated in R6/2 Mice and Normalized with LM11A-31

**Fig. 9:**
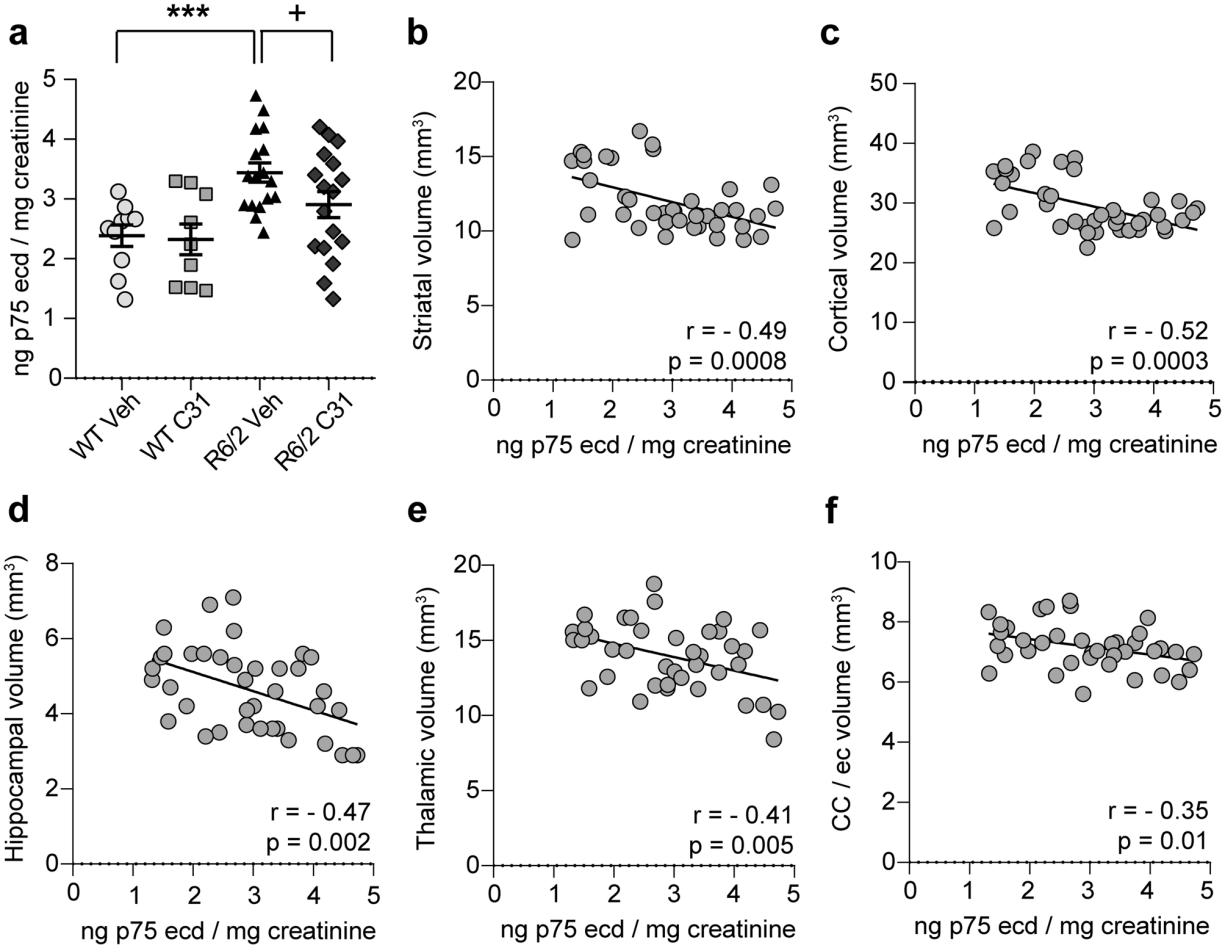
*Elevated urinary p75*^*NTR*^*-ecd levels are reduced with LM11A-31 treatment of R6/2 mice and negatively correlate with MRI-assessed regional brain volumes.***a** Urinary levels of p75^NTR^-ecd in WT and R6/2 mice treated with vehicle (Veh) or LM11A-31 (C31) (*n* = 9–17 mice/group) as measured with an ECL-based sandwich immunoassay on the MSD platform. Means (± s.e.m.) were from samples run in duplicate for each mouse and are expressed as ng p75^NTR^-ecd/mg creatinine. Statistical significance was determined with an ANOVA and Fisher’s LSD test. ***p = 0.0009 versus WT-Veh and ^+^*p* = 0.04 versus R6/2-Veh. **b**–**h** Scatterplots with linear regression lines showing the associations between urinary p75^NTR^-ecd levels and absolute ROI volumes calculated from T_2_-weighted MR images of the **b** striatum, **c** cortex, **d** hippocampus, **e** thalamus, and **f** corpus callosum (CC) with contiguous external capsule (ec). Experimental groups are combined for the analysis. Pearson correlation coefficients (*r*) and *p* values are shown.

Brain levels of p75^NTR^ are elevated with injury and neurodegeneration, including the striatum of HD patients and mouse models [7, 8, 10, 13]. Levels of the ecd of p75^NTR^ are also increased during neurodegeneration, as the receptor is cleaved with pro-apoptotic ligand binding [86], and can be excreted in urine as seen in ALS patients and mouse models [21, 87]. Thus, urinary p75^NTR^-ecd levels have been suggested as a biomarker for neurodegenerative diseases and may be particularly useful as a marker of treatment response for LM11A-31 as it is the target receptor for the ligand. We investigated this possibility here and found that levels of p75^NTR^-ecd were increased by 44 ± 7% in the urine of R6/2-Veh mice compared with WTs (Fig. 9a; Cohen’s *d* = 1.71). LM11A-31 treatment reduced these elevated levels by 14 ± 7% in R6/2 urine (Cohen’s *d* = 0.68) but did not affect levels in WTs. Interestingly, urinary p75^NTR^-ecd levels negatively correlated with all the absolute regional brain volumes calculated from T_2_-weighted MR images, except for the globus pallidus (*r* = − 0.15, *p* = 0.18), with the strongest correlations involving the striatum, cortex, and hippocampus (Fig. 9b–f).

### Machine Learning Approaches for Biomarker Selection from Multivariate Datasets

**Fig. 10:**
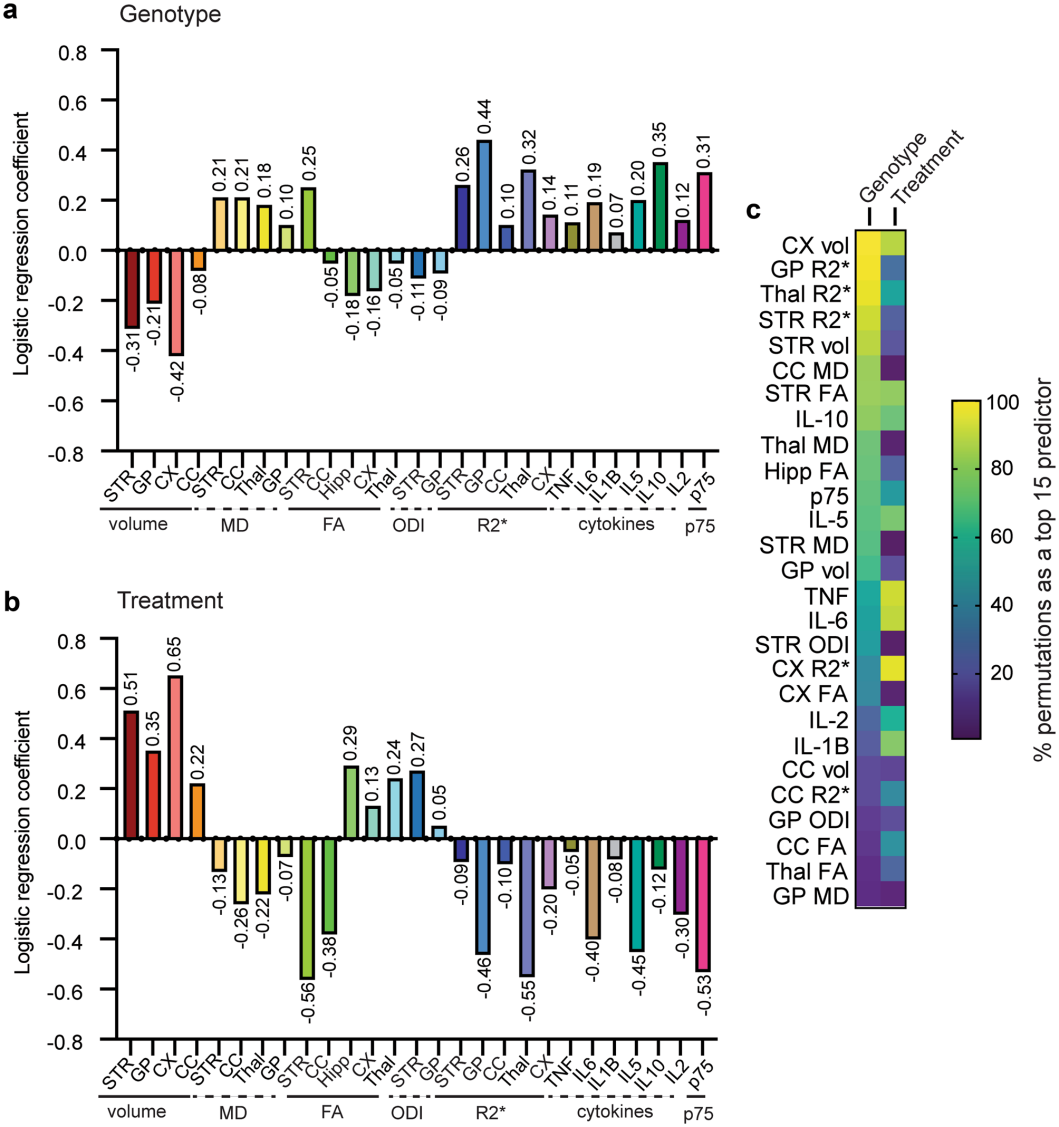
*Linear regression coefficients and feature importance heat map*. **a**, **b** Bar graphs of the linear regression coefficients of the 27 z-normalized features (neuroimaging or biofluid markers) that showed significant effects in standard statistical analyses were used as predictor variables for discriminating between **a** vehicle-treated WT and R6/2 mice (genotype) or **b** R6/2 mice given vehicle or LM11A-31 (treatment); *n* = 12–13 mice/group. For the genotype comparison, the WT-Veh group is coded as 0 and the R6/2-Veh group as 1, so positive coefficients are features positively associated with R6/2-Veh and negative coefficients are inversely correlated to the R6/2-Veh group. For treatment, R6/2-Veh group is coded as 0 and R6/2-LM11A-31 as 1. **c** Heat map showing the percentage of times the 27 features appeared as a top 15 predictor in the 1000 train-test permutations of the data subsets using the SVM-KNN model for genotype and the XGB-RFC model for treatment. Abbreviations: CC = corpus callosum; CX = cortex; FA = fractional anisotropy; GP = globus pallidus; Hipp = hippocampus; IL = interleukin; KNN = k-nearest neighbors; MD = mean diffusivity; ODI = orientation dispersion index; R2* = MR-based relaxometry; RFC = random forest classifier; STR = striatum; SVM = support vector machine; Thal = thalamus; TNF = tumor necrosis factor; vol = volume (by MRI); XGB = extreme gradient boosting.

Several of the markers investigated in this study showed statistically significant changes between genotypes and with treatment and boast large effect sizes (Table 1) suggesting they may be useful as biomarkers of disease state and therapeutic efficacy. Single markers have limited clinical relevance; thus, it would be useful to determine if combinations of these markers would be more efficacious. One way to address this question is to apply machine learning to evaluate multivariate predictions of genotype and treatment status. Machine learning approaches have been used to assess multidimensional datasets including those involving neuroimaging biomarkers in HD patients [20, 88]. Such analyses typically require large sample sizes for high accuracy and reliability. Although the subject number of the current study is small, machine learning can provide constructive information on variable importance, with the caveat that the findings need to be validated with a much larger sample size. Accordingly, a logistic regression model was used to analyze multiple z-normalized explanatory variables concurrently and to determine the magnitude of the association between these predictors and the response variables [44]. The 27 features (neuroimaging or biofluid markers) that showed significant effects in standard statistical analyses were used as predictor variables for discriminating between vehicle-treated WT and R6/2 mice (genotype) or R6/2 mice given vehicle or LM11A-31 (treatment). The features were sorted by their logistic regression coefficients and the five top predictors for genotype categorization were globus pallidus R2*, cortical volume (as assessed with MRI), plasma IL-10 levels, thalamic R2*, striatal volume (MRI), and urinary p75^NTR^-ecd levels (the latter two features were equal in magnitude; Fig. 10a). The five top predictors for treatment categorization were cortical volume, striatal FA, thalamic R2*, urinary p75^NTR^-ecd, and striatal volume (MRI) (Fig. 10b). Several features, namely striatal and cortical volume (MRI) and urinary p75^NTR^-ecd concentration, were in the top five predictors for both genotype and treatment and had large effect sizes (Table 1) suggesting these features may be particularly useful as composite biomarkers.

A more complex two-step machine learning approach was used to evaluate how many and which features are the most informative in combination as predictors of genotype or treatment. Feature selection using SVM or XGB determined importance which allowed for recursive feature elimination examining combinations of 2 to 27 features [47, 48]. Using two commonly used machine learning classifiers (KNN or RFC), we trained and then tested models for genotype or treatment prediction using the ranked feature sets [45, 46]. These models were each run on 1000 permutations of randomly chosen training and test subsets of the data. The resulting models from the four algorithm combinations (SVM-KNN, SVM-RFC, XGB-KNN, or XGB-RFC) were compared based on mean values of prediction accuracy, precision, and recall in classifying the test data (Suppl. Figs. 6 and 7). This analysis revealed that, for genotype classification, the SVM-KNN algorithm had the highest prediction accuracy, precision, and recall, and that ~ 17–20 features (biomarkers) can distinguish between the vehicle-treated WT and R6/2 mice with ~ 98% accuracy. The XGB-KNN and XGB-RFC algorithms have a 95% prediction accuracy using ~ 4–7 features. A therapeutic effect was discriminated with ~ 84% accuracy using ~ 11–14 features and the XGB-RFC algorithm. The 27 features were ranked according to the percentage of times they appeared as a top 15 predictor in the train-test permutations of the data subsets (Fig. 10c). Cortical and striatal volume (MRI) and R2* for globus pallidus, thalamus, and striatum were the five features that most frequently occurred (88–100%) as top predictors of genotype (i.e., occurred as a top 15 predictor in 88–100% of the 1000 permutations). The features occurring most frequently regarding treatment were cortical volume (MRI) and R2* as well as plasma levels of TNF-α and IL-6 and striatal FA (81–96%). The common features that occur in the top 15 when considering both genotype and treatment were MRI-assessed cortical volume, striatal FA, urinary p75^NTR^-ecd, plasma TNF-α and IL-5, and R2* for thalamus and globus pallidus. Again, given the small sample size and that these analyses require the data to be split into even smaller training and test subsets, these findings need to be validated in future experiments with larger datasets to improve accuracy and increase reliability.

## Discussion

The inability to translate promising preclinical disease-modifying therapeutic strategies to successful HD clinical trials may be remedied by developing pharmacodynamic biomarkers that are applicable from mouse-to-human. This study investigated the preclinical efficacy of multiple non-invasive techniques, including volumetric MRI, DTI, and biofluid assays, as potential pharmacodynamic biomarkers for HD using LM11A-31 as a prototype therapy with the expectation of translating these biomarkers to human clinical trials. Few, if any, preclinical HD studies use multiple biomarkers encompassing multi-modal *in vivo* neuroimaging in conjunction with fluid biomarkers and, many of these potential biomarkers have yet to be empirically tested for their efficacy in elucidating treatment response of putative HD therapeutics. The results of this study suggest that volumetric MRI, DTI, certain plasma cytokines, and urinary p75^NTR^-ecd may be used in tandem as markers of treatment response in HD. MRI-assessed volumes of striatum and cortex, striatal FA, and urinary p75^NTR^-ecd concentration were top predictors of genotype and treatment based on machine learning algorithms and had large effect sizes suggesting these features may be particularly useful as composite biomarkers.

The current structural MRI analysis indicated regional brain atrophy in the striatum, globus pallidus, cortex, and corpus callosum/ec of vehicle-treated R6/2 mice compared with WTs that was alleviated with LM11A-31 treatment. These data support previous structural MRI studies in HD patients and mice suggesting that regional brain volume reductions, particularly in striatum, are sensitive and reliable measures of disease state and/or progression (HD patient review: [18]; Mouse studies: [35, 38, 39, 49, 89–93]). A recent study using standardized analyses to compare imaging data from large scale prospective studies (PREDICT-HD, IMAGE-HD, and TRACK-HD) determined that volumes of the caudate, putamen, and globus pallidus had consistent large effect sizes across studies and provided greater statistical power than clinical markers [94], suggesting that, to date, structural MRI is the strongest candidate marker for use in clinical trials. The early detection of striatal atrophy via MRI in premanifest HDGCs, including young adults, means that neuroprotective treatments could start, and their effects may be tracked, as early as 15–20 years before symptoms manifest [18, 52, 95–99]. Despite much evidence showing the usefulness of MRI-detected striatal atrophy in monitoring HD state and progression, the current study and only a few other preclinical studies have demonstrated the feasibility of using *in vivo* MRI to detect or monitor the effects of neuroprotective agents in HD mouse models [91, 100–102]. The present study found that volumetric MRI represented some of the largest genotype and treatment effect sizes in HD mice compared with the other biomarkers examined, supporting its use as a promising pharmacodynamic biomarker to translate from preclinical to clinical studies.

Microstructural changes, as evidenced by DTI metrics FA and MD, often occur before brain atrophy and are sensitive to alterations at the cellular and molecular levels indicating brain connectivity changes that occur as fibers reorganize and/or degenerate [17, 18]. DTI studies of pre-symptomatic HDGCs and/or symptomatic HD patients demonstrated increased MD compared with healthy controls in multiple brain regions, including caudate, putamen, thalamus, hippocampus, and corpus callosum [17, 18, 53, 103–105]. In this study, each of these brain areas also showed elevated MD values in late-symptomatic R6/2 mice. MD is sensitive to gliosis, the density of axon bundles, and cell size and integrity; high MD values are associated with unrestricted water diffusion, reduced cellular membrane density, and white matter atrophy [18, 53]. LM11A-31 reduced MD to WT magnitude in the globus pallidus, the primary target of medial spiny neurons [106], and corpus callosum indicating it may prevent loss of neuronal integrity and/or axonal degeneration in these regions as it has been shown to prevent dendritic/axonal injury and p75^NTR^ can positively regulate myelination [8, 11, 107–109]. Concerning FA, diffusivity is reliably increased in the striatum and globus pallidum and reduced in the corpus callosum of premanifest HDGCs and manifest HD patients [17, 18, 53, 55, 103, 104, 110, 111]. In this study, R6/2 mice showed FA changes in the same directions as in HD patients in the striatum, hippocampus, and corpus callosum; LM11A-31 normalized these values in the former two areas, with the greatest effect size in the striatum. High FA values, as seen in the striatum, indicate that diffusion is mainly occurring along the primary fiber orientation and may arise as the striatum reorganizes after the loss of sub-cortical connections (i.e., striatopallidal) or from glial responses and increases in ferritin-bound iron due to neurodegenerative processes [61, 110, 112]. Taken together, LM11A-31’s normalizing effects on diffusivity suggest that it may alleviate disrupted connectivity and/or neuronal damage occurring before atrophy, although longitudinal studies are needed to test this hypothesis further.

NODDI can be more sensitive to gray matter changes than DTI metrics and can be used to interrogate key aspects of FA, including spatial orientation (ODI) of axons and dendrites [113, 114]. Until the present study, NODDI data in an HD mouse model or HD gray matter had yet to be evaluated. We found that ODI values were significantly reduced in the striatum with a trend toward a decrease in the globus pallidus and that ODI was restored to WT levels with LM11A-31 treatment. ODI decrements can indicate reduced fiber complexity and can occur if two fiber bundles cross and only one degenerates; thus, the striatal ODI decreases seen here may reflect the preferential degeneration of striatopallidal connections that occurs in HD [52, 110, 114]. ODI changes in gray matter have also been associated with secondary fiber degeneration, dendrite arborization pathology, and microglial density [113, 115]. Therefore, LM11A-31’s effects on ODI may reflect its alleviation of neurite degeneration of striatal cholinergic interneurons and microglial activation, as shown in our previous R6/2 studies [8, 16]. A NODDI study of premanifest HDGCs showed that neurite density and ODI were reduced in white matter regions (gray matter was not investigated) and that ODI correlated with UHDRS scores [52], suggesting that early axonal degeneration underlies white matter atrophy and contributes to disease severity. Thus, with more testing for reliability, NODDI metrics may improve the sensitivity and biological specificity of diffusion MRI and may have potential as HD biomarkers.

MR-based relaxometry in the current study showed that R2* values decrease in all brain regions examined, except the hippocampus, in R6/2 mice given vehicle and were normalized with LM11A-31 treatment. The R2* decrease was unexpected given the immunohistological evidence that ferritin was significantly increased in the R6/2 striatum in the current and previous reports [26] and that clinical studies showed that pre-symptomatic HDGCs and manifest HD patients have elevated R2* values in multiple brain areas, particularly striatum and globus pallidus, suggesting elevated iron content [57, 61, 116]. Similar findings in HD patients were obtained in more recent studies using QSM, which may confer more sensitivity than R2* [62–64]. QSM was not possible with our study due to technical factors. Increased iron content could reflect tissue atrophy if regional iron concentrations are elevated while total iron remains stable [64, 70]. Here we show that in R6/2 mice iron accumulation per volume is increased in each brain area examined, as seen in the basal ganglia of HD patients [57], and that LM11A-31 selectively ameliorates this elevation in the striatum. MR-based relaxometry is primarily responsive to ferritin-bound iron deposits, especially in basal ganglia structures [67, 68], however, other factors can influence R2* values. For example, R2* does not distinguish between high iron or calcium-containing structures, is affected by heterogeneous iron distribution, can be corrupted by background field gradient effects, and has a strong myelin dependence [64, 68, 117]. Future studies may control these inconsistencies by using QSM, which may be a more reliable and sensitive method for quantifying iron specifically and it is not as affected by microscopic iron distribution or field strength and imaging parameters [68, 117]. Although the absolute R2* values in the R6/2 striatum were incongruous with the elevated iron levels seen in HD patients and mice, this measure did show significant differences between R6/2 mice and WTs and was able to detect an effect of LM11A-31. Thus, MR-based relaxometry, especially QSM, may be useful as a treatment response biomarker, particularly if neuroinflammation or metal chelation including iron is being pursued as a potential therapeutic strategy [78, 118–120].

In addition to neuroimaging biomarkers, we investigated peripheral biofluid markers that have the potential to measure the response to therapeutics. Given the ample evidence that mHtt-induced neuroinflammation contributes significantly to HD pathogenesis, the peripheral immune response has been investigated as a source of putative biomarkers [121–124]. Here, we demonstrated that plasma levels of 6 of the 10 cytokines investigated were significantly elevated in R6/2 mice and that the increases in IL-6, TNFα, IL-1β, and IL-5 were ameliorated with LM11A-31 treatment. Notably, we previously showed that LM11A-31 also prevented elevated concentrations of IL-6 and TNFα in the R6/2 striatum and that these effects correlated with reduced microglial activation as assessed with TSPO-PET imaging [16]. Elevated cytokine and chemokine levels have been detected in the brain and plasma of HD patients and some increased significantly with disease progression [81, 84, 85, 125–127]. Of these cytokines, IL-6 showed the most reliable results with plasma elevations seen in 5 of 8 independent HD patient studies and was detected as early as 16 years before symptom onset, representing one of the earliest biochemical changes identified in HD [19, 81, 85, 121]. Plasma cytokine levels may also indicate other non-HD-related comorbidities that may arise particularly in late-stage patients; however, they may still be useful pharmacodynamic biomarkers in pre- and early-stage patients, particularly if used concurrently with other markers. Corroborating the current results, elevated plasma levels of IL-6 were reported in R6/2 and other HD mouse models, even at early to mid-symptomatic stages, and two other preclinical HD studies showed that potential therapies could normalize these levels highlighting the potential of plasma IL-6 as a translatable pharmacodynamic biomarker [81, 84, 85, 102, 128].

This study is the first to examine levels of p75^NTR^-ecd as a potential HD biomarker and showed large increases in urinary p75^NTR^-ecd in R6/2 mice. These increases negatively correlated with MRI-detected atrophy in the R6/2 striatum, among other brain regions, and therefore may be indicative of underlying neurodegeneration. Notably, elevated urinary p75^NTR^-ecd in R6/2 mice was alleviated by LM11A-31 suggesting that this measure may be effective as a marker of both disease state and treatment response in HD. In the brain, p75^NTR^ is up-regulated in the striatum of HD patients and mouse models and is its levels are normalized in preclinical studies with some putative HD therapeutics, including LM11A-31 [7–9, 13, 129]. LM11A-31 also increased other cleavage products of p75^NTR^ in R6/2 striatum, which is consistent with target engagement, and normalized signaling associated with the receptor in two HD mouse models [8]. Previous studies have shown that p75^NTR^-ecd levels are elevated in the urine of ALS patients and mouse models and altered in the blood (increased) and CSF (decreased) of Alzheimer’s disease patients and are thus being considered as prognostic and/or staging biomarkers in these diseases [21, 22, 87, 130]. This measure may be particularly useful for LM11A-31, which binds to p75^NTR^, as it could provide information on target engagement, or for other therapeutics that affect brain levels of p75^NTR^. The method of detecting p75^NTR^-ecd in mouse urine, which was developed in-house, is amenable to human studies, and to address translation feasibility, evaluation of urinary p75^NTR^-ecd levels in HD patients is underway in our laboratory.

The present study suggests that a multi-modality imaging approach combined with biofluid indices may be the most sensitive way to detect treatment response in HD preclinical and clinical studies as limitations exist for some of these biomarkers when used in isolation. Biomarkers that offer complementary advantages would be the most powerful option. For example, the use of volumetric MRI to assess striatal atrophy is one of the most sensitive imaging biomarkers in HD and, while detected in premanifest HDGCs, may rely on neuronal loss, which therapeutic intervention would precede ideally. DTI, functional MRI, and magnetic resonance spectroscopy may detect pathology at earlier stages than volumetric MRI, and NODDI affords sensitivity and better definition of the underlying neurobiological basis of pathology [19, 39, 92, 93, 131–135]. Thus, using these MRI metrics together could provide interrelated information and may reduce noise inherent to each measure. The biofluid biomarkers investigated here and in other studies (e.g., CSF and plasma neurofilament light protein [95, 124]) also offer technical advantages including quantification in high throughput assays, which confers reliability, and their ease of acquisition, particularly of plasma and urine, which makes their use feasible at all disease stages. Thus, each of these markers is potentially efficacious in detecting treatment response at multiple disease stages and using them contemporaneously, in addition to more established clinical outcomes for use at later disease stages (e.g., UHDRS), may be especially useful in clinical therapeutic intervention studies [136]. In support of this approach, machine learning techniques have shown that multiple types of MRI data can distinguish premanifest HDGCs from healthy controls [20, 88]. Machine learning methods were applied to the present dataset and identified several features with salient information for discriminating between genotypes and treatment response. Some of the identified features also had large effect sizes and included striatal and cortical volume, striatal FA, and urinary p75^NTR^-ecd concentration. Although these findings require validation with larger datasets, the classification models had good predictive accuracy and provide proof-of-concept that applying machine learning to multidimensional preclinical data may offer initial indications of biomarker combinations which could aid in clinical translation.

Biomarker validation studies would be most effective if conducted longitudinally starting before or at early symptom onset. It would also be advantageous for disease progression and treatment response biomarkers to detect neuropathological changes and neuroprotective effects at pre- or early symptomatic stages, as treatment may need to start at this time to achieve maximum benefits. A limitation of this study is that it employed a cross-sectional design and examined R6/2 mice at a late-disease stage. The R6/2 mouse model is useful as a time efficient first screen of a putative therapeutic’s neuroprotective effects in symptomatic preclinical trials as it has a rapid and reproducible phenotype [23–25]. However, the rapidity of disease progression and short lifespan renders early interrogation of treatment effects difficult. Ongoing and future studies in our laboratory aim to corroborate the present findings and address longitudinal changes in neuroimaging and biofluid markers using the Q175 knock-in mouse model, which express full-length mutant huntingtin and have a slower disease progression [93, 137].

In conclusion, we have provided preclinical evidence for the potential of several non-invasive biomarkers to detect pharmacological response and/or target engagement, the latter regarding LM11A-31 specifically as a p75^NTR^ ligand. These biomarkers closely relate to underlying HD pathology, a desired attribute of a biomarker, and many of them have been validated in large-scale longitudinal studies such as TRACK-HD and PREDICT-HD. Moreover, this study is the first to suggest levels of urinary p75^NTR^-ecd as a surrogate marker of disease state and therapeutic efficacy in HD. Thus, using volumetric MRI, DTI, and biofluid markers in tandem could be a feasible and powerful option to use as pharmacodynamic biomarkers for HD clinical trials. Given that LM11A-31 reduced HD phenotypes in multiple mouse models and is in a Phase 2a clinical trial for Alzheimer’s disease, the identification of these potentially translatable biomarkers able to detect its therapeutic effects meets a much-needed requirement for its advancement to HD clinical testing.

**Table 1:**
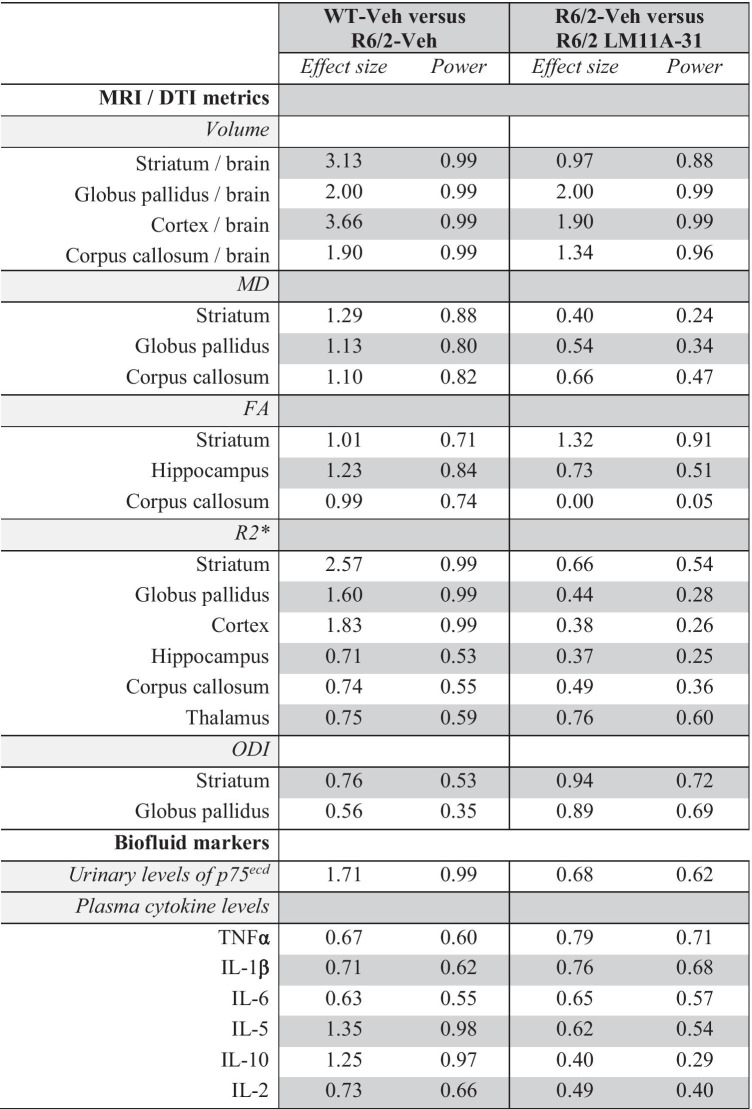
Genotype and treatment effect size and observed power of imaging metrics and biofluid markers

Effect size (Cohen’s *d*) and observed power were computed with G*Power software (α = 0.05). A *d* value of 1 indicates the two groups differ by 1 standard deviation and *d* = 0.8 is considered a large effect. Observed power (*i.e.* post-hoc power) is the probability of determining a significant effect of the test based on the effect size.

## Supplementary Information

Below is the link to the electronic supplementary material.

Supplementary file1 (PDF 543 KB)

Supplementary file2 (PDF 570 KB)

Supplementary file3 (PDF 570 KB)

Supplementary file4 (PDF 570 KB)

Supplementary file5 (PDF 579 KB)

Supplementary file6 (PDF 579 KB)

Supplementary file7 (PDF 579 KB)

Supplementary file8 (PDF 579 KB)

Supplementary file9 (PDF 570 KB)

Supplementary file10 (PDF 544 KB)

Supplementary file11 (PDF 552 KB)

Supplementary file12 Suppl. Fig. 1 Absolute regional volume reductions as assessed with MRI in WT and R6/2 mice at 11-12 weeks of age. (A-G) Volumes of the (A) striatum, (B) globus pallidus, (C) cortex, (D) dorsal hippocampus, (E) thalamus, (F) corpus callosum (cc) /contiguous external capsule (ec) and (G) whole brain were measured from T2-weighted MR images. ROI volumes were not adjusted for total brain size as in Figure 2. The ROIs and the whole brain were significantly smaller in R6/2 mice given vehicle (R6/2-Veh) compared to WT-Veh mice. LM11A-31 (C31) alleviated volume decreases in the striatum, globus pallidus, cortex and corpus callosum/ec. *n* = 8-16 mice/group. Results are expressed as mean ± s.e.m. Statistical significance was determined with an ANOVA and Fisher’s LSD with an FDR correction. **p = 0.0007 and ***p = 0.0001 versus WT Veh; +*p* ≤ 0.05 and +++*p* = 0.0005 versus R6/2-Veh (TIF 13055 KB)

Supplementary file13 Suppl. Fig. 2 LM11A-31 does not significantly affect mean diffusivity (MD) and fractional anisotropy (FA) in cortex and certain other sub-cortical ROIs of WT and R6/2 mice. (A-C) Mean diffusivity and/or (D-F) fractional anisotropy (FA) values in the (A, D) cortex, (B) hippocampus, (C, F) thalamus and (E) globus pallidus of WT and R6/2 mice with and without LM11A-31 (C31) treatment at 11-12 weeks of age. ROIs with significant changes in MD and FA are shown in Figure 4. *n* = 7-12 mice/group. Results are expressed as mean ± s.e.m. Statistical significance was determined with an ANOVA and Fisher’s LSD with an FDR correction. *p ≤ 0.05 versus WT-Veh (TIF 10116 KB)

Supplementary file14 Suppl. Fig. 3 Brain regions with no change in orientation dispersion index (ODI) between R6/2 and WT mice. (A-D) ODI in the (A) cortex, (B) hippocampus, (C) thalamus, and (D) corpus callosum (CC) with contiguous external capsule (ec) of 11-12 week-old WT and R6/2 mice given vehicle (Veh) or LM11A-31 (C31) (*n*=7-12 mice/group). Results are expressed as mean ± s.e.m. Statistical significance was determined with an ANOVA and Fisher’s LSD (TIF 7725 KB)

Supplementary file15 Suppl. Fig. 4 Mean relaxation rates (R2*) with ROI volume correction in WT and R6/2 mice with and without LM11A-31 treatment. (A-F) R2* values / ROI volume (mm³) in the (A) striatum, (B) globus pallidus, (C) cortex, (D) thalamus, (E) hippocampus, and (F) corpus callosum (cc) /contiguous external capsule (ec) of 11-12 week old WT and R6/2 mice given vehicle (Veh) or LM11A-31 (C31). Figure 6 shows mean R2* values without ROI volume correction. *n* = 7-15 mice/group. Results are expressed as mean ± s.e.m. Statistical significance was determined with an ANOVA and Fisher’s LSD or *t* test with an FDR correction. ***p = 0.0001, **p = 0.002, and *p = 0.008 versus WT-Veh; +*p* = 0.041 versus R6/2-Veh (TIF 10019 KB)

Supplementary file16 Suppl. Fig. 5 Associations between mean relaxation rates (R2*) and absolute ROI volume in WT and R6/2 mice. (A-F) Scatterplots with linear regression lines showing the associations between R2* values and absolute ROI volume (mm^2^) of the (A) striatum, (B) globus pallidus, (C) cortex, (D) hippocampus, (E) thalamus, and (F) corpus callosum (cc) /contiguous external capsule (ec). The experimental groups are combined for the analysis (*n*=7-15 mice/group). Pearson correlation coefficients (*r*) and *p* values are shown (TIF 7419 KB)

Supplementary file17 Suppl. Fig. 6 Prediction accuracy, precision and recall of the machine learning algorithms in genotype classification. Line graphs showing the accuracy (top graph), precision (middle), and recall (bottom) of the models in classifying new data by genotype using different numbers (*n*) of features from 2 to 27. The features are the 27 biomarker outcomes that were statistically significant between genotypes or with treatment. The algorithms used were support vector machines (SVM), extreme gradient boosting (XGB), k-nearest neighbors (KNN), and random forest classifier (RFC). Four machine leaning models were built with a feature importance and recursive feature selection algorithm followed by a classifier algorithm (SVM-KNN, SVM-RFC, XGB-KNN, or XGB-RFC). For each of the four models, the data was randomly split into training and test sets (*n*=6 mice /group in the test set, *n*=6-7 mice/group in the training set) for 1,000 permutations. Results are expressed as mean ± standard deviation. Accuracy indicates the proportion of correct classifications when *n* features are used as predictors. The precision is positive predictive value indicating model validity (high precision means less false positives) and recall indicates sensitivity (high recall means less false negatives). (TIFF 826 KB)

Supplementary file18 Suppl. Fig. 7 Prediction accuracy, precision and recall of the machine learning algorithms in treatment classification. Line graphs showing the accuracy (top graph), precision (middle), and recall (bottom) of the models in classifying new data by treatment using different numbers (*n*) of features from 2 to 27. The features are the 27 biomarker outcomes statistically significant between genotypes or with treatment). The algorithms used were support vector machines (SVM), extreme gradient boosting (XGB), k-nearest neighbors (KNN), and random forest classifier (RFC). Four machine leaning models were built with a feature importance and recursive feature selection algorithm followed by a classifier algorithm (SVM-KNN, SVM-RFC, XGB-KNN, or XGB-RFC). For each of the four models, the data was randomly split into training and test sets (*n*=6 mice/group in the test set, *n*=6-7 mice/group in the training set) for 1,000 permutations. Results are expressed as mean ± standard deviation. Accuracy indicates the proportion of correct classifications when n features are used as predictors. The precision is positive predictive value indicating model validity (high precision means less false positives) and recall indicates sensitivity (high recall means less false negatives). (TIF 67530 KB)
