# Supplementary figures and images for: Neuroimaging, Urinary, and Plasma Biomarkers of Treatment Response in Huntington’s Disease: Preclinical Evidence with the p75NTR Ligand LM11A-31
Source: Neurotherapeutics. 2021 Mar 30;18(2):1039–63. doi: 10.1007/s13311-021-01023-8 (PMC8423954; doi:10.1007/s13311-021-01023-8)

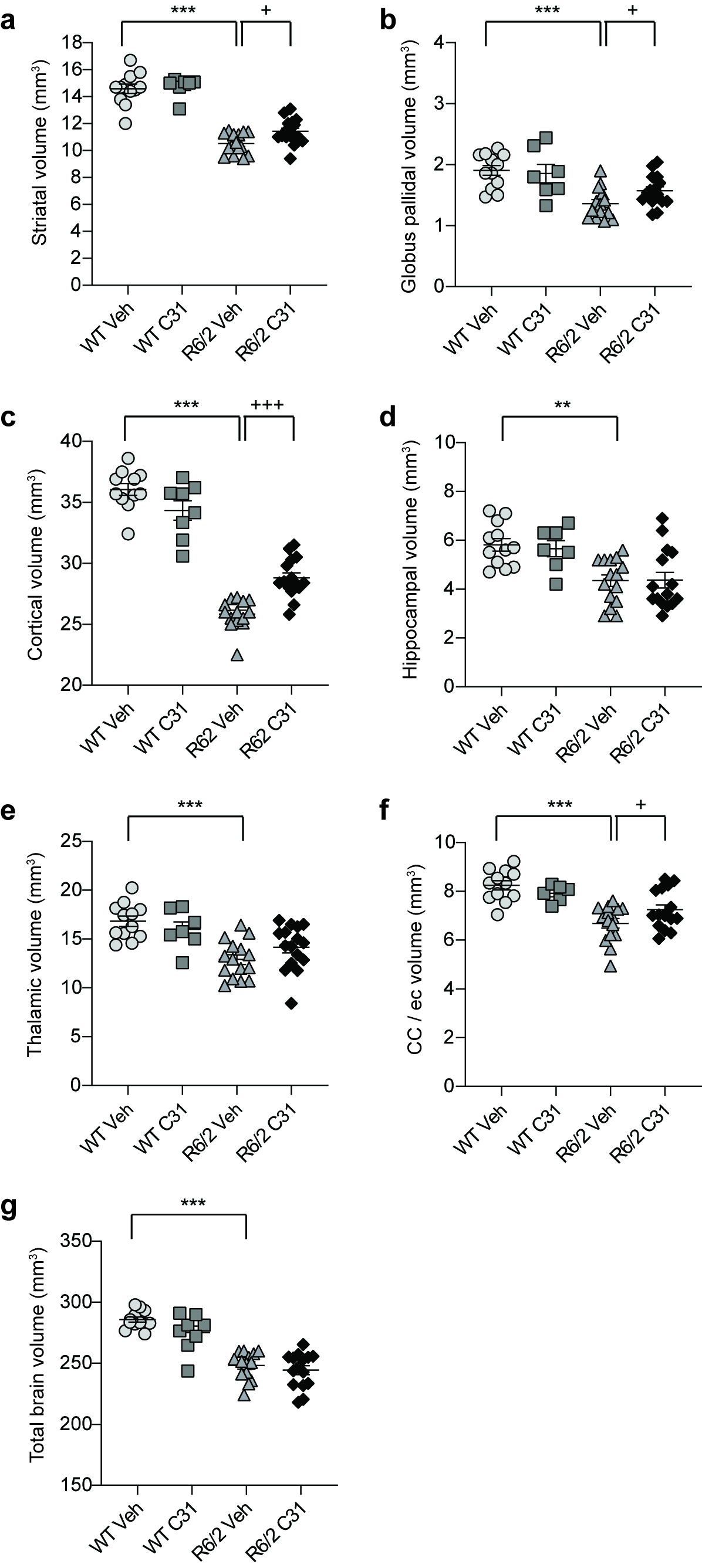

Supplement: Supplementary file 12 — Supplementary file12 Suppl. Fig. 1 Absolute regional volume reductions as assessed with MRI in WT and R6/2 mice at 11-12 weeks of age. (A-G) Volumes of the (A) striatum, (B) globus pallidus, (C) cortex, (D) dorsal hippocampus, (E) thalamus, (F) corpus callosum (cc) /contiguous external capsule (ec) and (G) whole brain were measured from T2-weighted MR images. ROI volumes were not adjusted for total brain size as in Figure 2. The ROIs and the whole brain were significantly smaller in R6/2 mice given vehicle (R6/2-Veh) compared to WT-Veh mice. LM11A-31 (C31) alleviated volume decreases in the striatum, globus pallidus, cortex and corpus callosum/ec. n = 8-16 mice/group. Results are expressed as mean ± s.e.m. Statistical significance was determined with an ANOVA and Fisher’s LSD with an FDR correction. **p = 0.0007 and ***p = 0.0001 versus WT Veh; +p ≤ 0.05 and +++p = 0.0005 versus R6/2-Veh (TIF 13055 KB) [file 13311_2021_1023_MOESM12_ESM.tif]

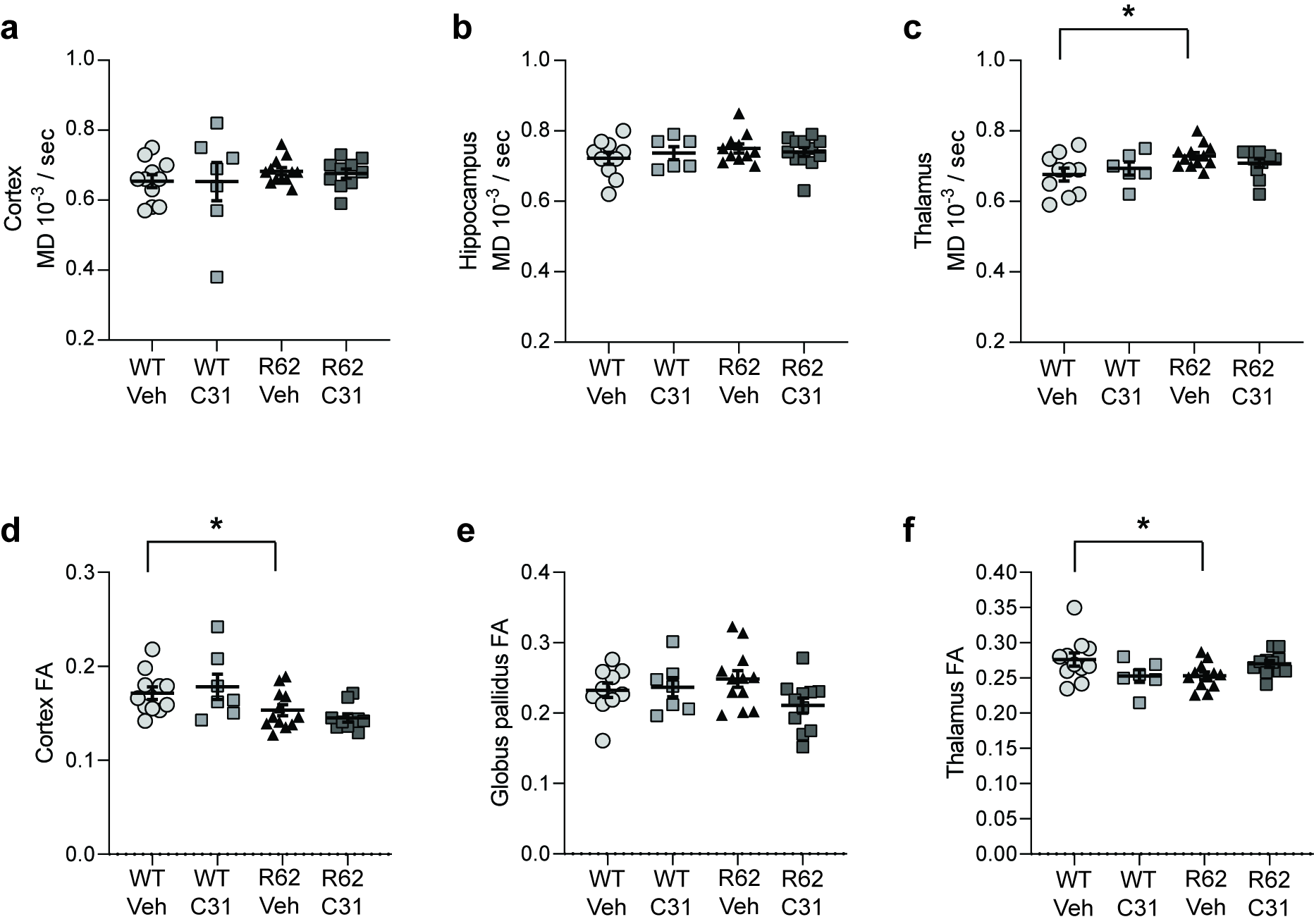

Supplement: Supplementary file 13 — Supplementary file13 Suppl. Fig. 2 LM11A-31 does not significantly affect mean diffusivity (MD) and fractional anisotropy (FA) in cortex and certain other sub-cortical ROIs of WT and R6/2 mice. (A-C) Mean diffusivity and/or (D-F) fractional anisotropy (FA) values in the (A, D) cortex, (B) hippocampus, (C, F) thalamus and (E) globus pallidus of WT and R6/2 mice with and without LM11A-31 (C31) treatment at 11-12 weeks of age. ROIs with significant changes in MD and FA are shown in Figure 4. n = 7-12 mice/group. Results are expressed as mean ± s.e.m. Statistical significance was determined with an ANOVA and Fisher’s LSD with an FDR correction. *p ≤ 0.05 versus WT-Veh (TIF 10116 KB) [file 13311_2021_1023_MOESM13_ESM.tif]

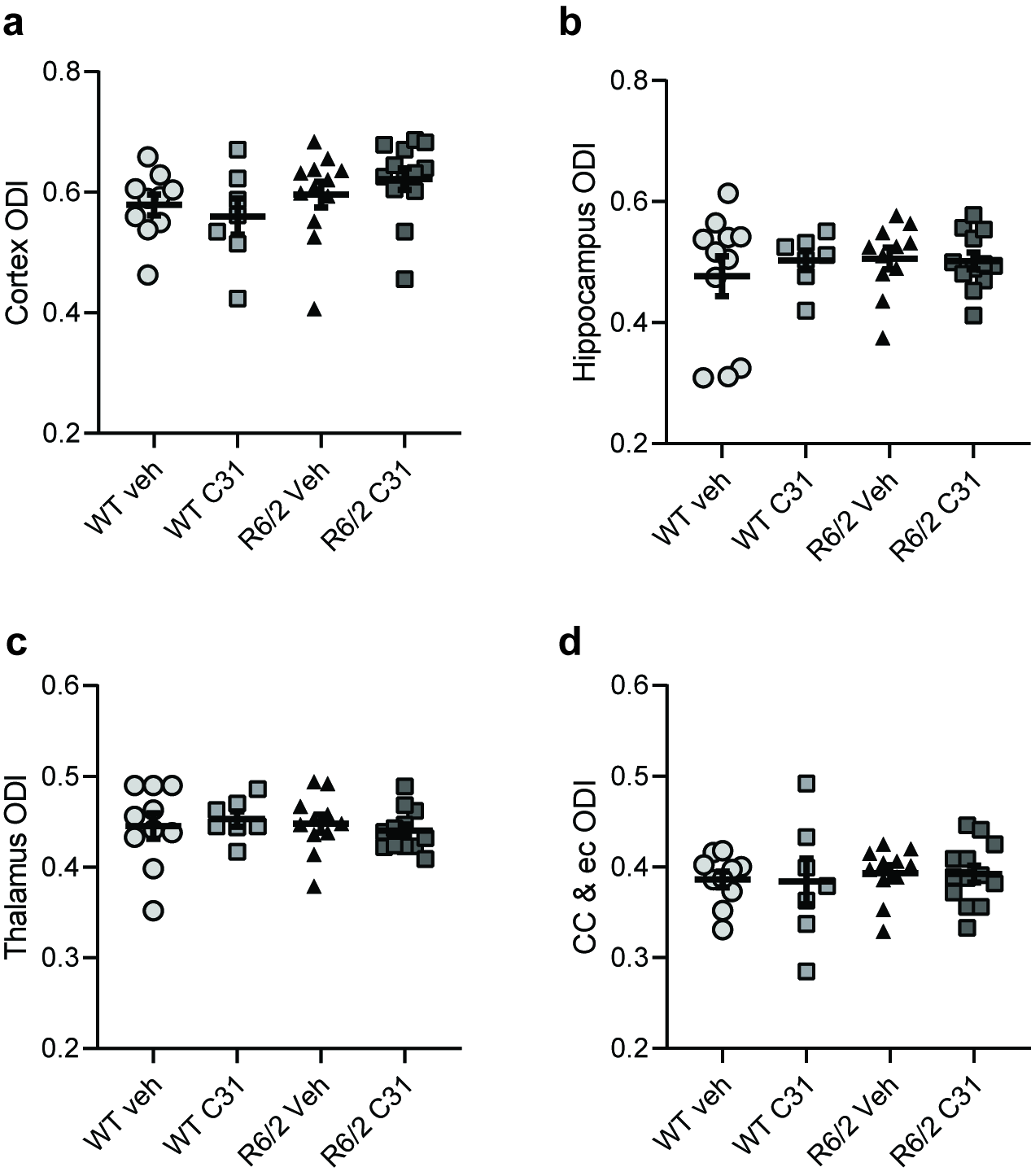

Supplement: Supplementary file 14 — Supplementary file14 Suppl. Fig. 3 Brain regions with no change in orientation dispersion index (ODI) between R6/2 and WT mice. (A-D) ODI in the (A) cortex, (B) hippocampus, (C) thalamus, and (D) corpus callosum (CC) with contiguous external capsule (ec) of 11-12 week-old WT and R6/2 mice given vehicle (Veh) or LM11A-31 (C31) (n=7-12 mice/group). Results are expressed as mean ± s.e.m. Statistical significance was determined with an ANOVA and Fisher’s LSD (TIF 7725 KB) [file 13311_2021_1023_MOESM14_ESM.tif]

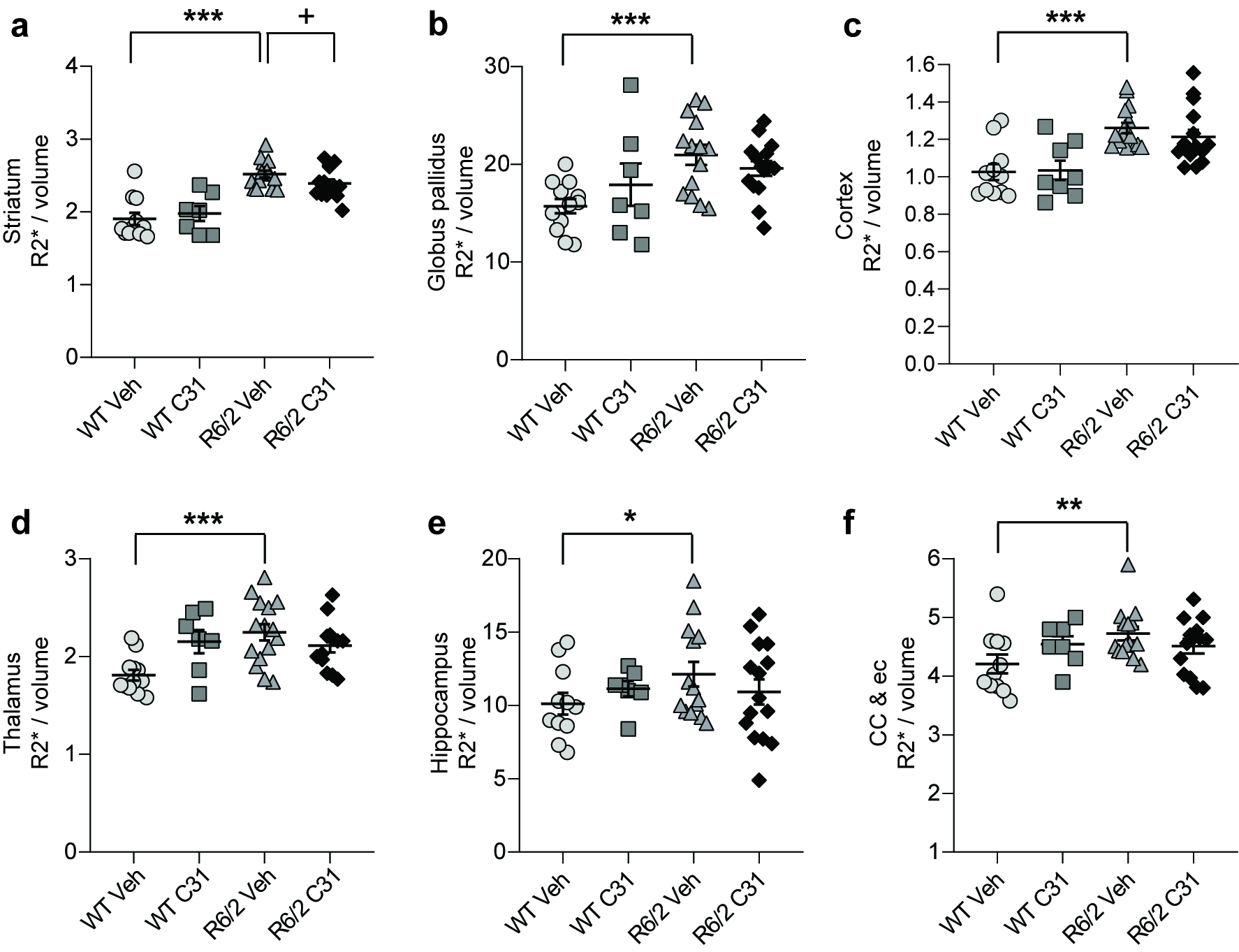

Supplement: Supplementary file 15 — Supplementary file15 Suppl. Fig. 4 Mean relaxation rates (R2*) with ROI volume correction in WT and R6/2 mice with and without LM11A-31 treatment. (A-F) R2* values / ROI volume (mm³) in the (A) striatum, (B) globus pallidus, (C) cortex, (D) thalamus, (E) hippocampus, and (F) corpus callosum (cc) /contiguous external capsule (ec) of 11-12 week old WT and R6/2 mice given vehicle (Veh) or LM11A-31 (C31). Figure 6 shows mean R2* values without ROI volume correction. n = 7-15 mice/group. Results are expressed as mean ± s.e.m. Statistical significance was determined with an ANOVA and Fisher’s LSD or t test with an FDR correction. ***p = 0.0001, **p = 0.002, and *p = 0.008 versus WT-Veh; +p = 0.041 versus R6/2-Veh (TIF 10019 KB) [file 13311_2021_1023_MOESM15_ESM.tif]

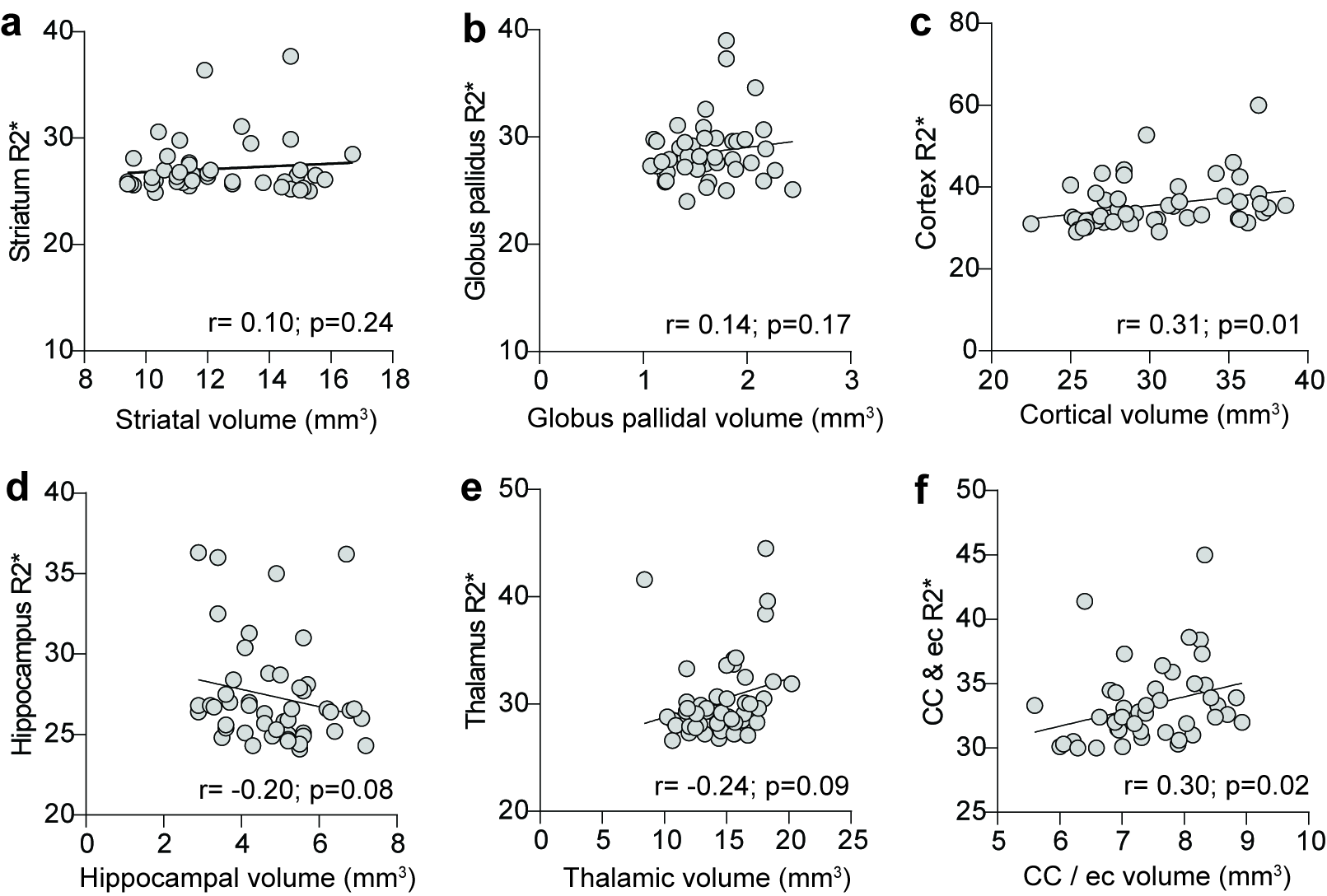

Supplement: Supplementary file 16 — Supplementary file16 Suppl. Fig. 5 Associations between mean relaxation rates (R2*) and absolute ROI volume in WT and R6/2 mice. (A-F) Scatterplots with linear regression lines showing the associations between R2* values and absolute ROI volume (mm2) of the (A) striatum, (B) globus pallidus, (C) cortex, (D) hippocampus, (E) thalamus, and (F) corpus callosum (cc) /contiguous external capsule (ec). The experimental groups are combined for the analysis (n=7-15 mice/group). Pearson correlation coefficients (r) and p values are shown (TIF 7419 KB) [file 13311_2021_1023_MOESM16_ESM.tif]

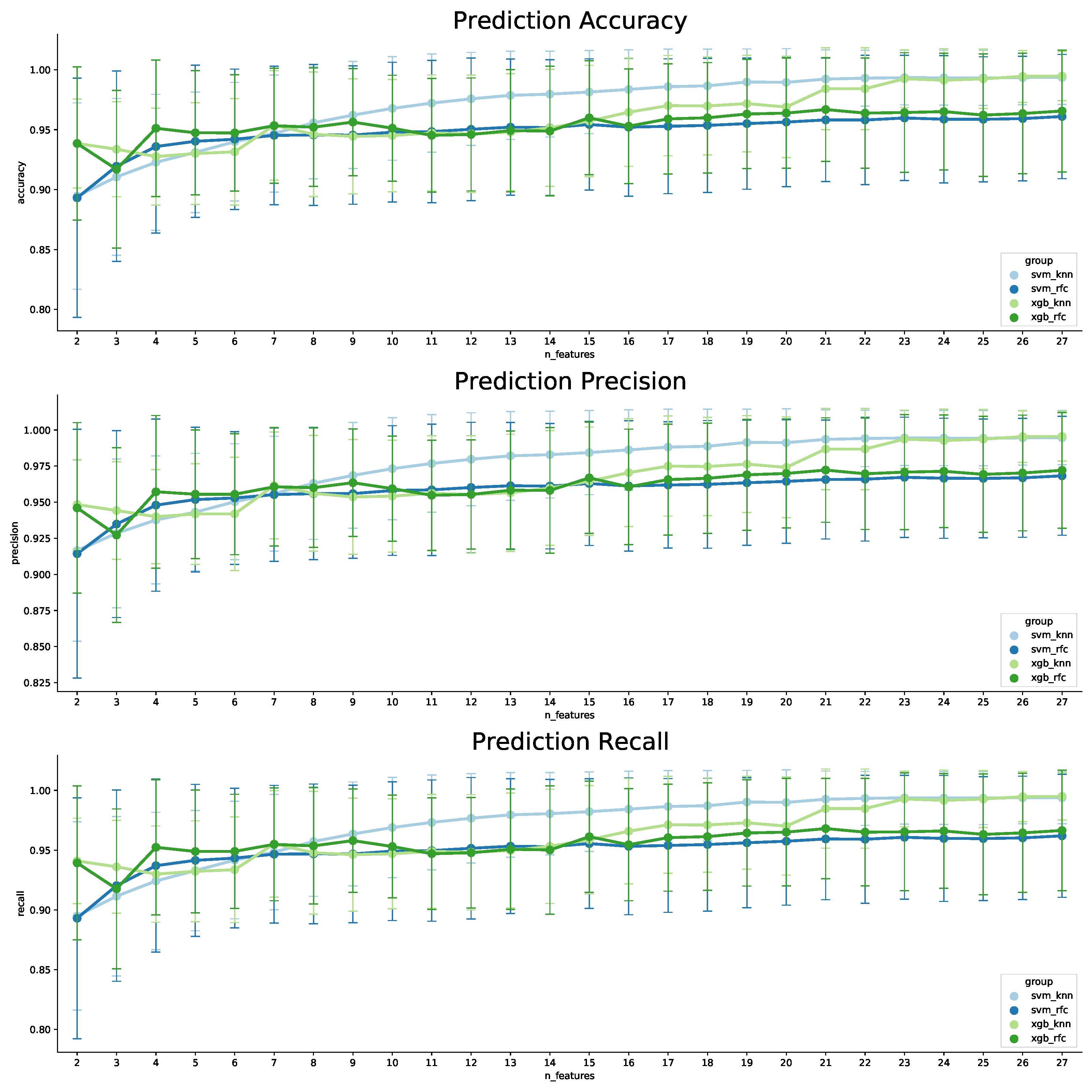

Supplement: Supplementary file 17 — Supplementary file17 Suppl. Fig. 6 Prediction accuracy, precision and recall of the machine learning algorithms in genotype classification. Line graphs showing the accuracy (top graph), precision (middle), and recall (bottom) of the models in classifying new data by genotype using different numbers (n) of features from 2 to 27. The features are the 27 biomarker outcomes that were statistically significant between genotypes or with treatment. The algorithms used were support vector machines (SVM), extreme gradient boosting (XGB), k-nearest neighbors (KNN), and random forest classifier (RFC). Four machine leaning models were built with a feature importance and recursive feature selection algorithm followed by a classifier algorithm (SVM-KNN, SVM-RFC, XGB-KNN, or XGB-RFC). For each of the four models, the data was randomly split into training and test sets (n=6 mice /group in the test set, n=6-7 mice/group in the training set) for 1,000 permutations. Results are expressed as mean ± standard deviation. Accuracy indicates the proportion of correct classifications when n features are used as predictors. The precision is positive predictive value indicating model validity (high precision means less false positives) and recall indicates sensitivity (high recall means less false negatives). (TIFF 826 KB) [file 13311_2021_1023_MOESM17_ESM.tiff]
